# Towards the development of a sustainable soya bean‐based feedstock for aquaculture

**DOI:** 10.1111/pbi.12608

**Published:** 2016-09-13

**Authors:** Hyunwoo Park, Steven Weier, Fareha Razvi, Pamela A. Peña, Neil A. Sims, Jennica Lowell, Cory Hungate, Karma Kissinger, Gavin Key, Paul Fraser, Johnathan A. Napier, Edgar B. Cahoon, Tom E. Clemente

**Affiliations:** ^1^ Department of Agronomy & Horticulture University of Nebraska‐Lincoln Lincoln NE USA; ^2^ Center for Plant Science Innovation University of Nebraska‐Lincoln Lincoln NE USA; ^3^ Department of Food Science and Technology The Food Processing Center University of Nebraska‐Lincoln Lincoln NE USA; ^4^ Kampachi Farms LLC Kona HI USA; ^5^ Centre for Systems and Synthetic Biology School of Biological Sciences Royal Holloway, University of London Egham Surrey UK; ^6^ Department of Biological Chemistry Rothamsted Research Harpenden Herts UK; ^7^ Department of Biochemistry University of Nebraska‐Lincoln Lincoln NE USA

**Keywords:** long‐chain Omega‐3 fatty acids, eicosapentaenoic acid, astaxanthin, soya bean oil, *Glycine max*

## Abstract

Soya bean (*Glycine max* (L.) Merr.) is sought after for both its oil and protein components. Genetic approaches to add value to either component are ongoing efforts in soya bean breeding and molecular biology programmes. The former is the primary vegetable oil consumed in the world. Hence, its primary usage is in direct human consumption. As a means to increase its utility in feed applications, thereby expanding the market of soya bean coproducts, we investigated the simultaneous displacement of marine ingredients in aquafeeds with soya bean‐based protein and a high Omega‐3 fatty acid soya bean oil, enriched with alpha‐linolenic and stearidonic acids, in both steelhead trout (*Oncorhynchus mykiss*) and Kampachi (*Seriola rivoliana*). Communicated herein are aquafeed formulations with major reduction in marine ingredients that translates to more total Omega‐3 fatty acids in harvested flesh. Building off of these findings, subsequent efforts were directed towards a genetic strategy that would translate to a prototype design of an optimal identity‐preserved soya bean‐based feedstock for aquaculture, whereby a multigene stack approach for the targeted synthesis of two value‐added output traits, eicosapentaenoic acid and the ketocarotenoid, astaxanthin, were introduced into the crop. To this end, the systematic introduction of seven transgenic cassettes into soya bean, and the molecular and phenotypic evaluation of the derived novel events are described.

## Introduction

Consumption of fish products has rapidly increased over the past 20 years and remains a major source of protein in the human diet worldwide. Previous estimates predicted over 50% of future fish harvest will be derived from aquaculture (Tidwell and Allan, 2001). These have been more refined and now reflect that by 2030, 62% of fish consumed will be derived from aquaculture, with 70% of the world's fish harvest demands emanating from Asia (World Bank, 2013). To meet this demand, innovations in areas encompassing evaluation of production practices (Henriksson *et al*., 2012), genetic enhancement of both species under culture (Clifford, 2014), and feedstocks sourced for aquafeed ingredients (Sissener *et al*., 2011) will be required. In regard to the latter, current feedstocks supplying protein and oil to the aquaculture industry are wild‐caught fisheries. While significant efforts are being put forth to properly manage the world's fisheries, the increasing demand placed on them is unsustainable (Naylor *et al*., 2000, 2008). Hence, alternative, renewable feedstocks will need to be developed. Terrestrial commodities are viable options to meet the demand for protein and oil in aquafeeds. When considering attributes a terrestrial feedstock should possess for the industry, soya bean (*Glycine max* Merr) is the one to prioritize. The rationale for soya bean is that it is one of the few commodities that partitions a significant amount of storage reserves as protein and oil, with approximately 40% and 20% of accumulation, respectively, in the mature seed. Secondly, soya bean is the most widely available feedstock for vegetable oil and quality protein on the world market and, lastly, being a legume, its nitrogen footprint is favourable. Moreover, the wealth of enabling technologies available to the crop, a complete draft genome sequence (Schmutz *et al*., 2010), genetic and physical maps (Shoemaker *et al*., 2008) and a reliable transformation system (Parrott and Clemente, 2004) make it ideal for targeted genetic improvements for output traits.

Towards this goal of developing a sustainable soya bean‐based feedstock for aquafeeds, a soya bean event was evaluated that produces an oil high in Omega‐3 fatty acids, containing approximately 30% each of α‐linolenic acid (ALA) and stearidonic acid (SDA; Eckert *et al*., 2006), in feed formulations for steelhead trout (*Oncorhynchus mykiss*) and Kampachi (*Serioloa rivoliana*).

A concomitant, complementary investigation to the feeding studies was the creation of soya bean lineages harbouring novel transgene stacks targeted for the synthesis of carotenoids coupled with Omega‐3 fatty acids in the oil. The seminal steps that laid the foundation to address the goal of designing terrestrial commodities for use in aquafeeds were the elucidation of the metabolic pathways governing the synthesis of very long‐chain polyunsaturated fatty acids (PUFA) along with the high‐value carotenoids and the translation of these findings in higher plants (Abbadi *et al*., 2004; Gerjets and Sandmann, 2006; Graham *et al*., 2007; Hasunuma *et al*., 2008; Jayaraj *et al*., 2008; Lu *et al*., 2010).

Exploiting results from these past endeavours, a genetic approach for the accumulation of the eicosapentaenoic acid (EPA) and astaxanthin in the seed of soya bean was designed. The information communicated herein describes an aquafeed formulation that drastically displaces fishmeal and fish oil in the diet of both freshwater and a high‐end finfish, without compromising growth parameters or nutritional quality of the flesh. Moreover, a genetic strategy for a prototype soya bean‐based feedstock for aquaculture, to add value to the oil, which theoretically should not negatively impact quality of the protein component of the seed, is communicated.

## Results

### The ability of steelhead trout (*Oncorhynchus mykiss*) to metabolize SDA to PUFAs

**Figure 1:**
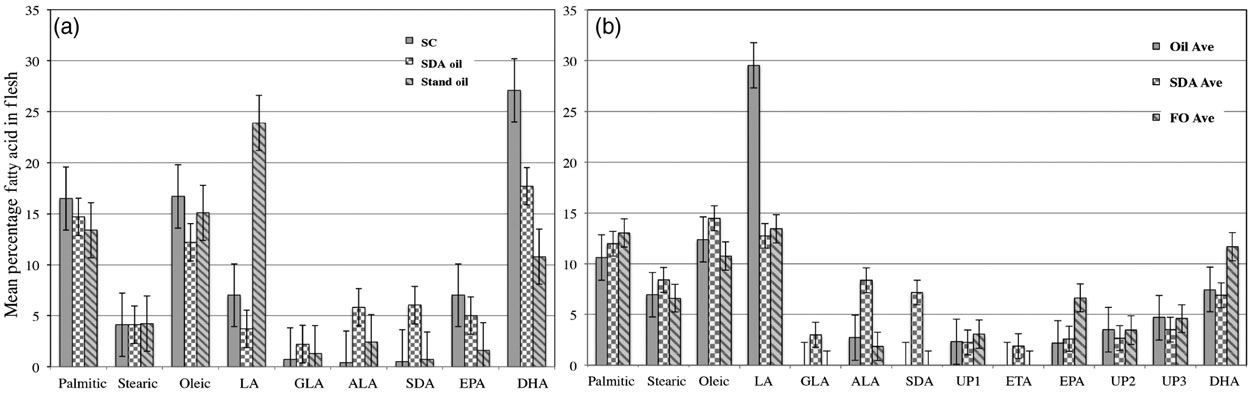
Fatty acid profile of flesh harvested from trout and Kampachi fed SDA oil‐coated feed pellets. The respective bars correspond to the mean fatty acid percentage (±SE) ascertained from flesh samples taken from trout (a) and Kampachi (b) fed the various test aquafeeds. Means derived from trout trial (a) were obtained from 16 flesh samples fed Silver Cup (SC) diet, 23 flesh samples fed commodity oil (Stand Oil) diet and 19 flesh samples fed SDA oil samples. Means derived from Kampachi trial (b) were obtained from 85 flesh samples fed standard oil‐coated pellets (Oil Ave), 86 flesh samples fed SDA oil‐coated pellets (SDA Ave) and 89 flesh samples fed fish oil‐coated pellets (FO Ave). Bars, left to right, within each panel, reflect means of corresponding legend designation, top to bottom. Fatty acid abbreviations are LA (linoleic acid), GLA (γ‐linolenic acid), ALA (α‐linolenic acid), SDA (stearidonic acid), ETA (eicosatetraenoic acid), EPA (eicosapentaenoic acid), DHA (docosahexaenoic acid). UP designates peaks observed that did not correspond to known standards.

The steelhead trout trial was designed to address the question whether *O. mykiss* can metabolize SDA to the PUFAs, EPA and DHA. To this end, a feed (Table S1; 100% SPC), devoid of marine ingredients was formulated. The extruded pellets were coated at a level of 13%, with either soya bean oil or SDA soya bean oil (Eckert *et al*., 2006). The amino acid profile of the soya bean protein concentrate (SPC) formulation is shown in Table S2. Thirty fish were fed per diet, with initial weight of each fish approximately 120 g. Following 90 days on the test aquafeeds, mean fish weights (g ± SDEV) were 263.4 ± 58.9, 247.9 ± 65.9 and 262.0 ± 49.4 for the SPC pellets coated with standard commodity soya bean oil, SDA oil and a commercial formulation, Silver Cup (app 42% protein, 16% oil), respectively. Fatty acid profile of the flesh was monitored across 23, 19 and 16 fish harvested from the SPC standard oil, SDA oil‐coated aquafeeds and commercial Silver Cup pellets, respectively (Figure 1a). Although there was a rather high variation of both EPA and DHA percentages within the sampled flesh, no significant variation between the Silver Cup and SPC‐formulated pellets coated with SDA oil was observed. However, there was a statistically significant drop in both EPA and DHA percentages when comparing the Silver Cup and SPC‐formulated pellets coated with commodity soya bean oil. Importantly, more total Omega‐3 fatty acids were present in the flesh samples derived from steelhead trout fed the SPC‐formulated pellets coated with the SDA soya bean oil (Figure 1a).

### Feeding trials with Kampachi (*Seriola rivoliana*) using SPC‐formulated aquafeed coated with SDA soya bean oil

The initial trial conducted with Kampachi mirrored the trout study wherein the tested diets were devoid of marine ingredients (100% SPC formulation, Table S1). The feed pellets were coated (19%) with SDA oil, commodity soya bean oil or fish oil. However, during 30‐day trial, mortality and emaciation were observed. Nevertheless, there was a sufficient number of fish within each feed batch to ascertain whether the Kampachi can metabolize SDA to EPA and DHA. The data on selected fish (10–15 samples) across the test feeds suggest that Kampachi only metabolizes SDA to eicosatetraenoic acid (ETA 20:4), the intermediate to EPA, which in turn led to reduction in accumulation of EPA and DHA (Figure 1b).

Given the poor performance of Kampachi fed the 100% SPC formulation, a subsequent study was conducted that evaluated formulations with SPC inclusion rates from 10 to 40% (Tables S1 and S2), with all feed pellets coated with fish oil. The results of this 77‐day feeding trial showed that displacement of greater than 10% fishmeal with SPC resulted in increased mortality, ranging from approximately 5% in commercial control and 0% SPC diets up to 16% in 30% SPC formulation, coupled with reduced growth rates.

**Figure 2:**
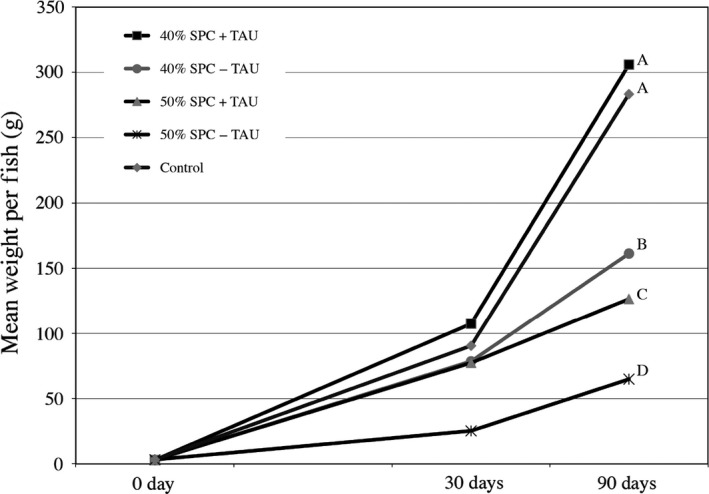
Impact of taurine supplementation to SPC‐formulated feed on Kampachi growth performance. Mean weight per fish (grams) was determined from a minimum of 45 fish per feed formulation per time point, sampled across three tanks. Feed formulation designations correspond to recipes listed within Table 1, with 40% SPC + TAU, 40% SPC − TAU, 50% SPC + TAU and 50%‐TAU as SPC‐40T, SPC‐40, SPC‐50T and SPC‐50 formulations, respectively. Control feed is the commercial diet Skretting. Final mean weights differentiated (*P* ≤ 0.01) using Develve software package.

These observations led to the evaluation of taurine supplementation in the SPC formulation with Kampachi, an amino acid previously demonstrated to be critical in aquafeed formulations with high inclusion of plant‐based proteins (Gaylord *et al*., 2006; Takagi *et al*., 2010), including the Kampachi relative *Seriola quinqueradiata* (Takagi *et al*., 2008). Here, formulations included 40% or 50% SPC supplemented with and without 4.6% taurine (Table 1). The 40% SPC formulation with taurine supplement (SPC‐40T) (Table 1) paralleled that of the commercial control (Skretting) diet, which contains approximately 0.5% taurine, with respect to growth rates (Figure 2). However, in agreement with the earlier trials, high inclusion rates of SPC, without taurine supplementation, translated to poor growth performance, and pushing the displacement of fishmeal further regardless of taurine supplementation in the feed, negatively impacted growth rates (Figure 2).

**Table 1:** Kampachi feed formulations

Component	SPC‐40	SPC‐40T	SPC‐50	SPC‐50T
SPC	40.00	40.00	50.00	50.00
Fish meal	11.89	11.89	1.89	1.89
Squid meal	7.42	7.42	6.06	6.06
Blood meal	4.40	4.40	4.40	4.40
Taurine	0.00	4.60	0.00	4.60
Cellulose	4.60	0.00	4.60	0.00
Soya lecithin	1.50	1.50	1.50	1.50
Vitamin premix‐F2	0.50	0.50	0.50	0.50
Stay‐C	0.06	0.06	0.06	0.06
Choline Cl	0.29	0.29	0.29	0.29
Mineral premix F‐1	0.25	0.25	0.25	0.25
Ca phosphate	1.50	1.50	1.50	1.50
Ca carbonate	0.01	0.01	0.01	0.01
L‐lysine	0.35	0.35	0.35	0.35
MHA^®^	0.38	0.38	0.38	0.38
Ethoxyquin	0.02	0.02	0.02	0.02
Mould inhibitor	0.02	0.02	0.02	0.02
Fish, HFPC	3.44	3.44	3.44	3.44

Component column list respective ingredients within the corresponding formulations. SPC – soya bean protein concentrate, Stay‐C refers to vitamin C, and MHA^®^ methionine source. Fish, HFPC refers to hydrolysed fish protein concentrate. Numbers within each of the formulation columns are percentage of corresponding ingredient. Pellets were coated with approximately 19% oil.

Given the positive productivity observed with the SPC‐40T formulation (Table 1), and the apparent inability of Kampachi to fully metabolize SDA to EPA and DHA (Figure 1b), as a means to further displace marine ingredients in the SPC‐40T formulation we evaluated an oil blend. A trial was carried out in which the SPC‐40T pellets were coated with either 50/50 blend of commodity soya bean oil/fish oil or SDA oil/fish oil and compared with a commercial aquafeed. The parameters and growth performance from this feeding trial were previously communicated (Lowell *et al*., 2011), herein additional information on the fatty acid profile of the harvested flesh from this trial (Table 2) is provided, wherein the salient point being that the SPC‐40T coated with 50/50 blend of SDA/fish oil translates to more total Omega‐3 fatty acids (ALA, SDA, ETA, EPA and DHA), in the harvested flesh as compared to the other feeds (Table 2).

**Table 2:** Fatty acid profile of Kampachi flesh fed SPC‐40T diet coated with soya bean oil/fish oil blends

SDA ratio	14:0	16:0	16:1	18:0	18:1	18:2	GLA	ALA	SDA	ETA	EPA	DHA
Skretting	1.7 ± 0.1B	17.0 ± 0.3	5.4 ± 0.2	6.0 ± 0.2	27.1 ± 0.6A	12.1 ± 0.4B	0.8 ± 0.6B	1.4 ± 0.2C	0.8 ± 0.3B	0.5 ± 0.0C	6.4 ± 0.2	7.6 ± 0.7
Soy/FO	2.5 ± 0.1A	15.0 ± 0.4	4.4 ± 0.2	5.2 ± 0.4	16.3 ± 0.6B	27.3 ± 0.8A	0.3 ± 0.0B	5.2 ± 0.2B	1.2 ± 0.0B	0.9 ± 0.0B	5.0 ± 0.2	6.7 ± 0.6
SDA/FO	2.5 ± 0.1A	15.2 ± 0.4	4.5 ± 0.1	5.4 ± 0.1	18.0 ± 0.8B	9.1 ± 0.1C	3.7 ± 0.1A	10.4 ± 0.2A	8.5 ± 0.2A	1.7 ± 0.1A	5.3 ± 0.2	6.4 ± 0.3

SDA ratio column indicates the oil blend coating, with Skretting diet serving as the commercial control in the feeding trial. Soy/FO refers to 50 : 50 blend conventional soya bean oil to fish oil and SDA/FO indicating SDA soya bean oil to fish oil. Numbers within the respective columns are means percentage of the respective fatty acid ± SD. Means were tabulated from flesh samples harvested from a minimum of nine fish. Abbreviations for fatty acids are as follows: 14:0 – myristic acid, 16:0 – palmitic acid, 16:1 – palmitoleic acid, 18:0 – stearic acid, 18:1 – oleic acid, 18:2 – linoleic acid, GLA – gamma‐linolenic caid, ALA – alpha‐linolenic acid, SDA – stearidonic acid, ETA – eicosatetraenoic acid, EPA – eicosapentaenoic acid, and DHA – docosahexaenoic acid. Columns with letters indicate significant differences among the means (*P* ≥ 0.01) as differentiated by Tukey's multiple comparison test. SPC‐40 feeds coated with 19% oil.

A subsequent trial was conducted to evaluate whether further displacement of fish oil in the SPC‐40T, 50/50 blend SDA/fish oil, will impact Omega‐3 fatty acid content of the flesh. Here, the SPC‐40T pellets (Table 1) were coated with either 50/50, 75/25 or 90/10 blend of SDA oil/fish oil. Mean fatty acid profiles of the harvested flesh are shown in Table S3. While total Omega‐3 fatty acids (ALA, SDA, ETA, EPA and DHA) averaged approximately 28%–30%, across the three oil blends, percentages of EPA and DHA drop by approximately 3% each in the fish fed pellets coated with the higher percentages of SDA oil (Table S3).

A grow‐out trial (238 days), evaluating the SPC‐40T 50/50 oil blend diet, was then carried out wherein 45 juvenile Kampachi were randomly placed in six 4‐MT tanks, translating to 270 fish total in the trial. Growth performance parameters from this study are shown in Table 3. No significant difference in feed conversion ratio (FCR) between the diets was observed. However, statistical changes were observed in tabulated means across parameters final weight, and daily weight gain, with the SPC‐40T formulation providing a boost in all parameters, under the conditions of this study (Table 3).

**Table 3:** Growth parameters tabulated for grow‐out feeding trial

Diet	MIW (g)	MFW (g)	MDG (g/day)	FCR
SPC‐40T	46.6 ± 1.9	1710.2 ± 119.7 A	7.0 A	1.4 A
Skretting	42.1 ± 0.7	1175.8 ± 12.9 B	4.8 B	1.3 A

Diet column indicates feed formulation (Skretting commercial control). MIW refers to mean initial weight of fish (grams), MFW indicates mean final weight (grams) ± standard deviation, MDG refers to mean daily growth (grams/day) and FCR indicates feed conversion ratio. Respective means within each column were separated by two‐sample *t*‐test (MiniTab Statistical Software, Minitab Inc State College, PA, USA).

**Figure 3:**
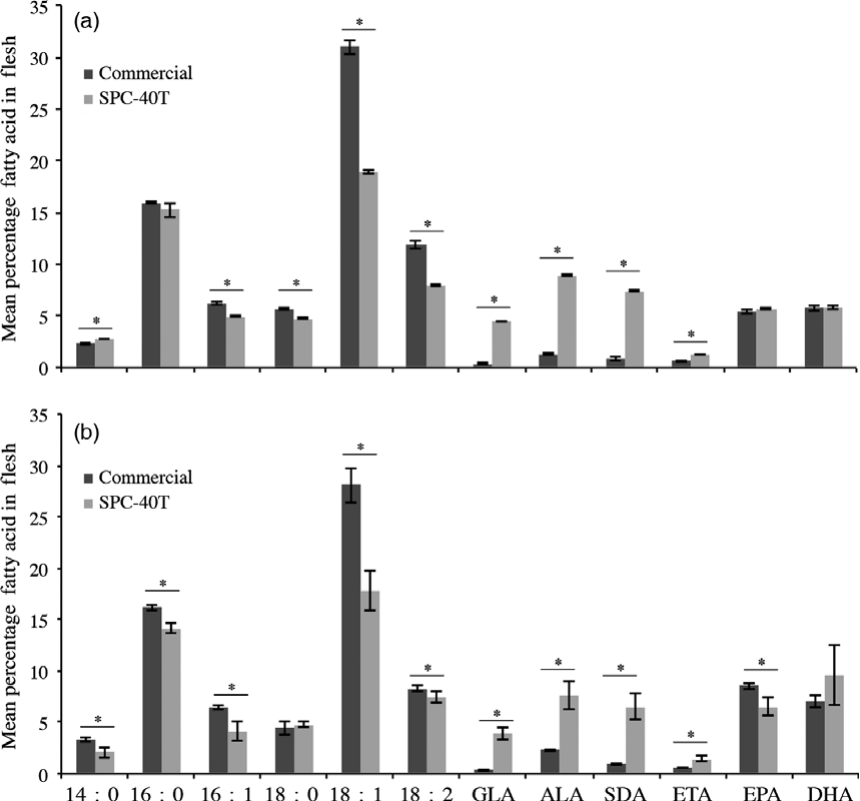
Fatty acid profile of flesh harvested from Kampachi during grow‐out trial on SPC‐40T formulation coated with SDA/fish oil blend. Panel 3a: Mean (±SD) fatty acid profile of flesh from 27 fish per formulation harvested following 78 days of feeding. Panel 3b: mean (±SD) fatty acid profile of flesh from 10 fish per formulation harvested following 278 days of feeding. The commercial diet with Skretting and SPC‐40T formulation (Table 1) was coated with a 50 : 50 blend of SDA:fish oil. Fatty acid abbreviations are 16:1 (palmitoleic acid), LA (linoleic acid), GLA (γ‐linolenic acid), ALA (α‐linolenic acid), SDA (stearidonic acid), ETA (eicosatetraenoic acid), EPA (eicosapentaenoic acid), DHA (docosahexaenoic acid). Means for each fatty avid were differentiated using unpaired *t*‐tests (*P* ≤ 0.01), GraphPad Prism v 6 (www.graphpad.com). Bars with * designate significant difference between means.

Fatty acid profile of tissues samples taken following 78 and 238 days of feeding was ascertained. The analyses revealed the SPC‐40T coated with 50/50 blend of SDA/fish oil did not compromise either EPA or DHA at both sampling dates (Figure 3). Moreover, in the fish fed SPC‐40T diet, more total Omega‐3 fatty acids were present in the flesh at both time points (Figure 3).

### Prototype soya bean feedstock for aquaculture

Two genetic constructs were assembled to create a prototype soya bean feedstock for aquaculture, targeting synthesis of two aquafeed ingredients, EPA and the carotenoid astaxanthin. The T‐DNA elements of the vectors designated pPTN809D5 and pASTA are shown in Figure S1.

Progeny derived from 15 and 21 events from pPTN809D5, and pASTA, respectively, were characterized at the molecular level. Among the pPTN809D5 events, two were carried on for further characterizations, designated 824‐1, 824‐8 (a clone of 824‐1) and 826‐4.

Molecular analysis of the selected pPTN809D5 events is shown in Figure S2. The fatty acid profile changes triggered by the dual expression of the introduced fatty acid elongase (Agaba *et al*., 2004) and ∆5 desaturase (Kinney *et al*., 2011) showed accumulation of the elongation products of oleic acid, linoleic acid and linolenic acid, namely eicosenoic acid, eicosadienoic acid and eicosatrienoic acid, respectively (Table S4).

### Stacking of fatty acid elongase and ∆5 desaturase with event 539‐5

Event 824 clones were crossed with event designated 535‐9 (Eckert *et al*., 2006). Fatty acid profiles of F_1_ seeds derived from this four‐gene stack led to the accumulation of EPA ranging from approximately 1% up to approximately 4% in the seed, and its immediate precursor, ETA, was present at approximately 11% (Table S5). Monitoring of F_3_ populations revealed the relative level of EPA in the seed was maintained over two generations; however, the major Omega‐3 fatty acid present in the oil was ETA (Table S6), with fidelity of the expression of all four transgenes demonstrated in F_2_ immature seed (Figure S3).

A subset of the events carrying the T‐DNA of pASTA (Figure S1) was selected based on yellowish coloration of the mature seed (Figure S4). These events were subsequently characterized at the molecular level (Figures S5 and S6), revealing that in some events not all transgenes were truly integrated. For example, event 806‐14 only carries the phytoene synthase (*Psy*), event 806‐12 carries the *Psy* and *CrtZ*, while event 807‐5 harbours *Psy* and *CrtW* (Figure S5). Northern analysis on the pASTA events was conducted (Figure S6). As expected, event 806‐14 only expressed *Psy*, event 806‐12 expressed both *Psy* and *CrtZ*, while event 807‐5 expressed both *Psy* and *CrtW*. However, the majority of events screened accumulated transcripts corresponding to the three transgenes (Figure S6).

Quantification of astaxanthin and β‐carotene was ascertained from 21 events (Figure S7). Event 806‐14, which only carries *Psy* cassette (Figures S5 and S6), accumulated β‐carotene at just over 800 μg/g seed, while event 807‐2, which co‐expresses the gene stack (Figure S6), accumulated astaxanthin to app. 25 μg/g seed, with a concomitant production of β‐carotene at 500 μg/g seed (Figure S7). Phenotyping of events that differentially expressed *CrtZ* or *CrtW* in conjunction with *Psy* revealed accumulation of other carotenoids downstream of β‐carotene. These include echinenone, canthaxanthin, phenicoxanthin and α‐carotene (Table S7).

Field plots were planted to monitor accumulation of carotenoids in the seed under field conditions. While these plots were too small to ascertain agronomic parameters, no obvious impact on germination, lodging and seed numbers per plant was observed. Levels of β‐carotene and astaxanthin present in the field production are shown in Figure S8, which tended to mirror that observed from the previous glasshouse‐grown harvest.

Given expression of *Psy* is expected to pull carbon downstream towards carotenoids and therefore compete for the substrate geranylgeranyl diphosphate (GGPP) towards tocopherols, it was hypothesized that shifting carbon flux down to carotenoids would reduce tocopherol accumulation in the oil. However, monitoring of tocopherol levels in the seed did not reveal a trend correlating tocopherol levels with carotenoid accumulation (Figure S9). Interestingly, accumulation of the three forms of tocotrienol was observed in the transgenic events, which in soya bean this form of vitamin E is not present (Figure S9).

To create the prototype soya bean‐based feedstock for aquaculture, which simultaneously accumulates astaxanthin and EPA, we crossed event 535‐9 (Eckert *et al*., 2006) with events carrying pPTN809D5, and pASTA T‐DNA elements, thereby assembling a seven gene‐of‐interests stack. Fatty acid profiles on coloured F_1_ seed were ascertained (Table S8), with coloured F_2_ generation profiles and carotenoid levels shown in Table S9. The levels of ETA and EPA, combined, ranged from approximately 3%–5% of the oil, along with astaxanthin and β‐carotene accumulation from 23 to 44.8 μg/g and 527 to 1139 μg/g seed, respectively.

Field trials were conducted in 2012 and 2013 with the pASTA events, 806‐14, 818‐13 and 807‐2, along with four‐gene stack linage for EPA synthesis, and four lineages harbouring the seven‐gene stack for the simultaneous production of carotenoids and PUFAs. While some variation in both fatty acid profiles and carotenoid levels across the 2 years were observed (Tables S10 and S11), data mirrored what was observed under glasshouse conditions for the respective events/lineages (Table S9).

## Discussion

Given its two primary coproducts, high‐quality protein and oil, the soya bean is an ideal target for the creation of a terrestrial feedstock for aquaculture. In order for the former coproduct to be fully maximized as a displacement for fishmeal in aquafeed formulations, especially for marine fish species, supplementation with the amino acid taurine (Espe *et al*., 2012; Gibson Gaylord *et al*., 2007; Rhodes *et al*., 2011; Watson *et al*., 2013), including the Hawaiian yellowtail relative, *S*. *quinqueradiata* (Takagi *et al*., 2008, 2010), is required. However to meet the goal of aquafeed devoid of fishmeal, taurine supplementation alone will not suffice, given the observation that inclusion of SPC beyond the SPC‐40T recipe was not sufficient to counteract negative growth performance of Kampachi (Figure 2).

The feeding trials herein targeted displacement of fishmeal and fish oil in a systematic fashion. We investigated inclusion of an SDA soya bean oil in aquafeeds for both freshwater and marine species, in which the former was capable of metabolizing SDA to EPA and DHA (Figure 1a). This result is contrary to what was observed with the nonanadromous rainbow trout, which were fed diets in which fish oil was displaced with echium oil wherein significant reductions in EPA and DHA in the harvested tissues occurred (Cleveland *et al*., 2012). A possible reason for the different outcomes, aside from potential genetic variation between the trout lineages, is echium oil contains approximately 12% SDA and 33% ALA (Berti *et al*., 2007); however, the Omega‐3‐enriched soya bean oil utilized herein possesses 30% of each 18 carbon Omega‐3 fatty acid (Eckert *et al*., 2006).

However, in regard to the latter, SDA was elongated to the fatty acid ETA, suggestive that *S. rivoliana* is lacking the fatty acid desaturation step to metabolize further (Figure 1b). A similar observation was seen in Atlantic salmon fed a SDA soya bean oil, Soymega^™^, wherein ETA is synthesized, and SDA accumulates, but with a reduction in EPA and DHA in a 50% FO lipid‐blended diet (Nanton *et al*., 2012).

These studies add to the literature on utility of terrestrial feedstocks for the development of sustainable aquafeeds, which have been genetically enhanced for synthesis of novel output traits. Such traits require some level of identity preservation to capture value (i.e. Soymega^™^), and ideally with yields comparable to elite germplasm. Albeit there are exceptions, in regard to identity preservation, for example in the case of high oleic acid soya bean developed through down‐regulation of the ∆12 fatty acid desaturase alone or in combination with a silenced palmitoyl thioesterase, stacked with mutant alleles of FAD3, being marketed under trade names Plenish^™^ and Vistive Gold^™^, respectively, should command a sufficient market demand to ultimately become the commodity soya bean.

Given the significant costs associated with identity preservation, coupled with the enormous expense to surmount regulatory hurdles of transgenically derived traits, production of multiple output traits through genetic stacking of alleles is an avenue that can enhance the economic attractiveness of a identity‐preserved required product. An example is the prototype soya bean‐based feedstock for aquaculture described here, where two output traits, an Omega‐3 fatty acid‐enriched oil, coupled with carotenoid synthesis. In regard to the latter, astaxanthin, with annual sales upward of $200 million (Li *et al*., 2011), commanding a $250 up to $1000 per kg market value, is a coproduct that can greatly enhance attractiveness of a identity‐preserved soya bean. This ingredient is incorporated in many feeds including, salmon, trout and shrimp (Higuera‐Ciapara *et al*., 2006; Hussein *et al*., 2006). A renewable source would be highly recommendable as presently chemical synthesis from petrochemical by‐products is the production method of choice.

A three‐gene stack introduced into soya bean was sufficient to accumulate from 25 to 38 μg/g seed astaxanthin (Table S9, Figure S7). The variation in astaxanthin level observed is likely due to environment, with a latter glasshouse propagation leading to an increased level of the carotenoid. Considering the lower range observed, 25 μg/g seed accumulation, approximately 4–6 million acres of such soya bean would need to be identity‐preserved to meet the estimated annual consumption of 100 000 kg of astaxanthin used in feed and nutraceutical applications. Hence, further optimizations of genetic designs are likely required, so to reduce plantings, and thus improve economic viability.

In soya bean, the only carotenoid detected in seed is lutein (Table S7) and is relatively low, under 10 μg/g seed. Expression of *Psy* alone (event 806‐14) led to the accumulation of β‐carotene up to approximately 800 μg/g seed, with a 29% reduction in lutein. Transgenic events in which co‐expression of the three‐gene stack led to enhanced flux to astaxanthin, reduction in endogenous lutein levels dropped by 50%. Although zeaxanthin was not detected, among total carotenoids in soya bean samples, β‐carotene predominated, with minimal accumulation of echinenone (Table S7), the ketolating product of β‐carotene, is suggestive that ketolation of β‐carotene was inefficient. Therefore, a possible route to increase astaxanthin content may be through targeting ketolase activity, a step previously considered as a limit for astaxanthin production *in planta* (Zhong *et al*., 2011).

The four‐gene stack assembled for the targeted synthesis of EPA led to accumulation of up to 5%, with total Omega‐3 fatty acid levels exceeding 60% (Table S6). While this change in fatty acid profile is impressive, to maximize value as an aquaculture feedstock, EPA levels will need to be improved, to better mirror fish oil, which tend to range from 9% to 15%.

The remaining percentage of SDA in the gene‐stacked seed suggests that elongation step was inefficient (Tables S5 and S6). Earlier attempts to produce EPA and DHA *in planta* via Δ6 destaurase/elongase pathway also resulted in low accumulation of EPA. This was attributed to poor exchange of Δ6‐desaturated acyl group between the acyl‐PC and acyl‐CoA pools (Abbadi *et al*., 2004; Qi *et al*., 2004; Venegas‐Calerón *et al*., 2010). Indeed, this metabolic bottleneck once bypassed via the incorporation of an acyl‐CoA‐dependent ∆6 desaturase from the alga *Ostrococcus tauri* resulted in high levels of accumulation of both EPA and DHA (Ruiz‐López *et al*., 2014).

This study demonstrates that a metabolic engineering strategy can be implemented that will lead to dual production of critical aquafeed ingredients in soya bean. Soya bean as such a platform is highly desirable due to its attributes as a legume, along with its well‐established cropping system and its historical utility in feed applications. Importantly soya bean, with annual worldwide production of 270 million tonnes, has the inherent capacity to displace the 21 million tonnes of fisheries harvest (FAO, 2014) that enters aquaculture industry for feed utilization in a sustainable, cost‐effective fashion.

While some biological hurdles remain, exploitation of the tools of synthetic biology (Collins, 2012; Facchini *et al*., 2012; Jenkins *et al*., 2011) will permit a facile strategy for the assembly of an array genetic cassettes linked on a single T‐DNA or an DNA element suitable for direct DNA delivery, incorporating a diverse set of promoter and terminator elements for optimal coordination of gene expression. The stacks created here were assembled by crossing, resulting in duplication of many genetic elements, along with multiple copies of the selectable marker cassette. Transcript profiling on the three‐ and four‐gene stacks did not suggest silencing of the stacked transgenic cassettes (Figures S3 and S6); however, duplication of genetic elements, and lack of attention to cassette orientation, is not ideal for stability of the phenotype over generations (Bhullar *et al*., 2003; Gudynaite‐Savitch *et al*., 2009; Singer *et al*., 2011, 2012; Yang *et al*., 2005). Moreover, investigations into metabolic networks leading to the synthesis of these two targets, EPA and carotenoids, have led to the identification alternative routes that have potential to enhance the accumulation of the desired products (Cunningham and Gantt, 2011; Petrie *et al*., 2010; Ruiz‐López *et al*., 2012; Venegas‐Calerón *et al*., 2010). Hence, through combination of optimal genetic designs, exploitation of synthetic biology and novel metabolic engineering strategies, a soya bean‐based feedstock for aquaculture is technically feasible.

## Conclusions

Aquaculture will help mitigate food security challenges over the next 50 years. Underpinning a sustainable avenue to attain the expected demand for farmed fish harvest is novel feed formulations that are not reliant upon wild fisheries for protein and oil. A logical approach resides in the development of terrestrial sources for aquafeed ingredients. Herein, a series of feeding studies that demonstrate such a strategy implementing a soya bean with a novel oil output trait to simultaneously displace fishmeal and fish oil in aquafeeds. Furthermore, as a means to enhance the attractiveness of a soya bean that has the potential to be a major constituent in aquafeed formulations, devoid of marine ingredients, a multitransgene stack approach for the concomitant synthesis of high‐value coproducts was demonstrated, serving as prototype terrestrial‐based feedstock to provide quality protein and oil, for the aquaculture industry.

## Experimental procedures

### Aquafeed preparation

The aquafeed diets were extruded at the Bioprocessing and Industrial Value Added Program (BIVAP) facility (Kansas State University, Manhattan, KS) or the Food Processing Center (University of Nebraska‐Lincoln, Lincoln, NE). The BIVP facility was used for preparation of the trout feeds. The dry ingredients containing particles larger than 1 mm diameter were mixed into a blend and hammer‐milled through a 3/64″ screen by Lortscher Agri Service, Inc. (Bern, KS). The remaining oversized ingredients needed to adjust the formulations were milled onsite at KSU using a Fitz mill (model D; Fitzpatrick Co., Elmhurst, IL) with a 1‐mm screen. Feed was extruded utilizing a Wenger X20/E325 single‐screw extruder system (Wenger Manufacturing, Inc., Sabetha, KS) including a gravimetric feeder, a conditioner, single‐screw extruder barrel, die and knife assembly. The blended dry formulation was fed at a metered rate from the gravimetric feeder into the conditioner section of the system. In the dual shaft‐equipped conditioner, the blend was conveyed and mixed while being hydrated and heated with a combination of water and steam prior to entering the extruder barrel. Inside the 10 : 1 L/D single‐screw extruder barrel, the formulation was further processed by the addition of water and mechanical kneading/shear and formed through a die to create an extrudate with properties sufficient to produce a 2‐ and 4‐mm durable sinking feed pellet. The speed of a rotating knife assembly was adjusted to maintain a cut length equivalent to the feed diameter. The cut feed was pneumatically conveyed to a Wenger gas‐fired belt dryer (Model 4800 series; Wenger Manufacturing, Inc.) where it was dried to shelf‐stable conditions.

The dried base feed was vacuum coated with an amount of test oil to bring the total lipid content of the finished feed to approximately 19%. The vacuum coating process was accomplished using a custom‐built rotating drum system (University of Nebraska‐Lincoln). The coating process involved pouring the measured amount of oil over a specified amount of the base feed. The tumbling drum was then sealed, a vacuum of 15 in Hg was drawn, and the drum was allowed to rotate for 5 min before finally releasing the vacuum. This vacuum tumbling process was repeated as necessary to force the oil inside the feed pellets. Fatty acid analysis of SDA/FO 50 : 50 used to coat feed was 4.0% (14:0); 13.4% (16:0); 5.6% (16:1); 3.6% (18:0); 12.9% (18:1), 6.6% (18:2); 3.4% (GLA); 11.1% (ALA); 12% (SDA); 0.7% (ETA); 6.4% (EPA); and 5.5% (DHA). Commercial feed fatty acid profile was similar with exceptions, 22.3% (18:1); 10% (18:2); 0.2% (GLA); 3.3% (ALA); 1.4% (SDA); 8.8% (EPA); and 7.6% (DHA).

The Food Processing Center was used for the preparation of the aquafeeds used in the Kampachi trials. The dry ingredients, containing particles larger than 1 mm diameter, were milled using a hammer mill (model #20SSHMBD; CS Bell Company, Tiffin, OH) fitted with a screen size of 0.7 mm. The dry ingredient formulations were blended in a ribbon mixer (model #; Wenger Manufacturing, Inc.) for 5 min. These prepared formulations were then processed into feed using a Wenger twin‐screw extrusion system consisting of a gravimetric feeder, Dual Diameter Conditioner (DDC), the twin‐screw extruder barrel and die/knife assembly (Wenger Manufacturing Inc.). The Wenger TX‐57 corotating twin‐screw extruder with a 25.5 : 1 L/D ratio was used with a screw configuration and processing conditions suitable for sinking aquatic feed production. Die insert sizes were selected to produce a finished feed of 2–9 diameters to better match the average size of the fish throughout the feeding trial. As the extrudate exited the die, a rotating knife assembly cut the pieces to the desired length. After cutting, the extrudate was pneumatically conveyed to a Wenger gas‐fired conveyor dryer (Model 4800 series; Wenger Manufacturing, Inc.), where it was dried to shelf‐stable conditions.

The prepared feed lipid content was adjusted, after extrusion, by varying the amount of blended oil content applied during a vacuum coating process. The oil used in the finished feed obtained from several sources. The fish oil (menhaden oil stabilized with 1000 ppm Coviox T‐50, Virginia Prime Gold) used was sourced from the Omega Protein Corporation (Houston, TX). The soya bean oil was cold‐pressed from whole soya beans using a screw‐type oil press (AgOilPress LLC, Eau Claire, WI). The pressed soya bean oil was stabilized with the addition of 200 ppm tert‐butylhydroquinone (TBHQ) and 200 ppm citric acid (anhydrous) and then stored in a 4 °C cooler until it was needed for coating. The dried base feed was vacuum‐coated with a blended amount of oil to bring the total lipid content of the finished feed to approximately 19%. The vacuum coating process was accomplished using the same vacuum coating system and procedure as described above.

### Feeding trials

The trout studies were conducted at Blue Valley Aquaculture (Sutton, NE, USA). Fish were fed 3.0‐mm formulated pellets, one tank per feed type. Feeding was conducted once per day, for 90 days, with equal volumes of respective feed provided per tank.

*Seriola rivoliana* feeding trials were carried out at Kampachi Farms (Kona, HI, USA). Trials were carried out in 4‐MT high‐density polyethylene tanks, populated with 48 juvenile fish per tank, derived from a single cohort. Average fish weight was determined for each tank during stocking. All tanks were supplied with flow‐through, ambient‐temperature filtered sea water with flow rates of 45 L/min. Tanks were cleaned daily. Fish were allowed to acclimate in tanks for 2 days prior to feeding trial. Test feeds were randomly assigned to each tank, with a minimum of three tanks per feed, and feeding was done by hand to visible satiation. Feeding rates changed based on pellet size needs, as reflected by fish growth. All uneaten pellets were collected and counted 30 min after feeding. The amount of feed consumed each day was tabulated.

Average weight gain was ascertained by final average fish weight – initial fish weight, and average daily growth determined by final average fish weight – initial average fish weight/number of days in trial. Feed conversion ratio was tabulated as average individual feed intake (dry weight)/average individual live weight gain.

### Construction of binary vectors

A bifunctional fatty acid elongase cDNA (gift from A.J. Teale) (Agaba *et al*., 2004), and a cDNA of a ∆5 fatty acid desaturase gene from *Saprolegnia diclina* (Kinney *et al*., 2011) were assembled into seed‐specific plant expression cassettes under control of the soya bean β‐conglycinin promoter. The respective elements were subsequently subcloned into the binary vector pPTN200, which carries a bar gene cassette (Thompson *et al*., 1987) under control of the Pnos promoter from *Agrobacterium tumefaciens*. The resultant binary vector is designated pPTN809D5 (Figure S1a).

A three‐gene stack was designed in a separate binary vector referred to as pASTA. Here, the β‐carotene hydroxylase (CrtZ), β‐carotene ketolase (CrtW) genes were from *Brevundimonas* sp., along with the maize phytoene synthase gene placed under control of seed‐specific promoters, and terminated by either the soya bean glycinin terminator or common bean transcriptional terminator (Figure S1b).

### Soya bean transformation

The respective binary vectors, pPTN809D5 and pASTA, were mobilized into *A*. *tumefaciens* strain EHA101 (Hood *et al*., 1986) and NTL_4_/pKPSF2 (Luo *et al*., 2001), respectively, via triparental mating. The derived transconjugants were used for soya bean transformations as previously described (Xing *et al*., 2000; Zhang *et al*., 1999) using the soya bean genotype Thorne (McBlain *et al*., 1993). Primary transgenic events were established and grown to maturity under glasshouse conditions.

### Molecular characterization of transgenic soya bean events

Southern blot analysis on transgenic events was carried out as previously described (Buhr *et al*., 2002). Total genomic DNA was isolated from young fully expanded leaves following a modification of the protocol out lined by Dellaporta *et al*. (1983). Ten micrograms of genomic DNA was restriction‐digested with either *Eco*R I, pASTA events or *Pst* I, pPTN809D5 events, and subsequently separated on 0.8% agarose gel. The separated DNAs were transferred to a nylon membrane (Zeta Probe GT; Bio‐Rad, Hercules, CA) by capillary transfer and fixed via UV cross‐linking. Probes were prepared by random prime synthesis incorporating dCT^32^P using Agilent Technologies picked up this product Prime‐It II kit following the manufacturer's protocol (Cat #300385). Membranes were hybridized in 1 mm EDTA, 0.5 m Na_2_HPO_4_ (pH 7.2), 7% SDS, 1% BSA at 65 °C overnight. Membranes were subsequently washed twice in 5% SDS, 40 mm Na_2_HPO_4_ solution for 30 min each, followed by a 30‐min wash in 1% SDS, 40 mm Na_2_HPO_4_ solution at 65 °C. For reprobing, membranes were stripped in 0.1 N NaOH, 0.2% SDS solution for 20 min at room temperature and washed twice in 0.2 m Tris (pH 7.5), 0.1× SSC, 0.2% SDS solution for 20 min at 95 °C.

Northern blot analysis was carried out on immature embryos as described by Buhr *et al*. (2002). RNA was extracted using TRIzol reagent (Cat #10296) following manufacturer's instructions. Fifteen micrograms of RNA was separated on a 1% formaldehyde agarose gel and transferred to a nylon membrane. Hybridization and washing conditions were performed as described above. Membranes were exposed to X‐ray film at −80 °C to visualize signals.

### Fatty acid analysis

Fatty acid analysis was conducted on soya bean and fish samples following the procedures outlined by Butte (1983) or Cahoon *et al*. (2001). Fatty acid profiles on the derived methyl esters were monitored on a 6890N gas chromatography–flame ionization detector (Agilent Technologies, Santa Clara, CA) fitted with a 30‐m X 250 μm HP‐INOWAX column (Cat # 19091N‐133; Agilent Technologies). Fatty acids are reported as percentages of total fatty acids.

### Carotenoid analysis

Total lipids were extracted from mature seed by the Bligh and Dyer method (Bligh and Dyer, 1959). Extractions were conducted on 5 ‘coloured’ seeds per plant, with 5 μg of trans‐beta‐apo‐8′‐carotenal added to 50 mg ground sample as an internal standard for quantification. Three millilitres of methanol/chloroform (2 : 1) was added and incubated at room temperature for 30 min, followed by the addition of 1 ml chloroform and 1.8 ml water and mixed. The mixture was subsequently centrifuged for 2 min at 1050 g, and the lower organic phase was transferred to a new tube. The previous steps were repeated twice, and the organic layer dried under N_2_ and dried sample resuspended in 3 ml acetone. The suspension was briefly centrifuged, and 1 ml of the top layer was used for HPLC analysis. HPLC analysis was carried out on an Agilent 12000 series system, fitted with a prontosil 200‐5‐C30 column. The elution was carried out with methanol: t‐butyl methyl ether (80 : 20, v/v) solvent system at room temperature, with a flow rate of 1.4 ml/min. Identification of carotenoids was based on co‐chromatography and comparative UV/Vis spectral characteristics with authentic standards. In addition to relative quantification to internal standard, dose–response curves were created for accurate quantification of individual carotenoid components.

### Tocopherol and tocotrienol analysis

Powder derived from soya bean samples was spiked with the internal standard 5,7‐dimethyl tocopherol, in a 5 μg per 50 mg powder ratio. The powdered mixture was then combined with 600 μl methanol/dichloromethane (9 : 1), gently mixed and incubated at room temperature for 30 min. The mixture was subsequently centrifuged for 5 min at 454 g, and 35 μl of the upper layer subjected to HPLC analysis. HPLC analysis was conducted on an Agilent technologies 1200 series system, fitted with a Eclipse XDB‐C18 column, eluted with a two‐solvent system, methanol/water (19 : 1) at a flow rate of 1.5 ml/min.

### Creation of gene stacks

For the targeted production of EPA, a cross was made using two pPTN809D5 events designated 824‐1 and 824‐8, with a transgenic soya bean event that produces high levels of stearidonic acid (SDA) referred to as 535‐9 (Eckert *et al*., 2006), which carries the borage ∆6 fatty acid desaturase gene (Sayanova *et al*., 1997), and the *Arabidopsis* ∆15 fatty acid desaturase gene, thereby creating a four‐gene stack in soya bean.

Lineages of the derived four‐gene stack producing EPA were subsequently crossed with pASTA events previously determined to producing carotenoids, 818‐3, 807‐2 and 806‐14. This in turn manifested various multigene stacks for the simultaneous production of EPA and carotenoids.

## Author contribution

H.P. and F.R. conducted soya bean transformations and molecular and phenotypic characterizations of derived transgenic events. S.W. designed formulations and prepared aquafeeds. N.A.S, J.L., C. H., C.K. P.A.P. and K.K. designed, conducted and analysed data from Kampachi feeding trials. P.F. carried out our carotenoid analyses. J.A.N and E.B.C. contributed to genetic designs for oil modification and assisted in lipid analyses of transgenic events. T.E.C. wrote the paper and coordinated activities of the project. The authors declare no conflicts of interest.

## Supporting information


**Figure S1** T‐DNA elements of pPTN 809D5 and pASTA binary vectors.
**Figure S2** Southern blot analysis on selected pPTN809D soya bean events.
**Figure S3** Northern blot analyses on four‐gene stack soya bean lineage derived from SDA event (535‐9) × 824‐1 event (pPTN809D).
**Figure S4** Phenotypic coloration of soya bean seeds and derived oil from selected pASTA events.
**Figure S5** Southern blot analyses on selected transgenic soya bean events (pASTA).
**Figure S6** Northern blot analyses on selected immature T_2_ generation embryos obtained from selected pASTA soya bean events.
**Figure S7** Astaxanthin and β‐carotene levels in selected transgenic soya bean (pASTA) seed grown under greenhouse conditions.
**Figure S8** Astaxanthin and ß‐carotene levels in selected transgenic soya bean (pASTA) seed grown under field conditions.
**Figure S9** Tocopherol and tocotrienol levels in selected transgenic soya bean (pASTA) seed grown under greenhouse conditions.
**Table S1** Aquafeed test formulations with incremental SPC inclusion rates
**Table S2** Amino acid profiles of aquafeed SPC test formulations
**Table S3** Fatty acid profile of Kampachi flesh fed diets with increasing percentage of SDA
**Table S4** Fatty acid profiles on T_1_ seed derived from pPTN809D5
**Table S5** Fatty acid profiles on F_1_ populations
**Table S6** Fatty acid profiles on F_3_ seeds producing EPA
**Table S7** Relative percentage of carotenoids in seed of selected transgenic soya bean events
**Table S8** Fatty acid profiles on selected orange colour F_1_ seeds producing ALA, SDA or EPA
**Table S9** Fatty acid profiles, total oil and carotenoid content on selected orange colour F_2_ seeds producing EPA
**Table S10** Fatty acid profiles and carotenoid content of mature soya bean seed from field harvest (2012)
**Table S11** Fatty acid profiles and carotenoid content of mature soya bean seed from field harvest (2013)
