# Supplementary material for: Towards the development of a sustainable soya bean‐based feedstock for aquaculture
Source: Plant Biotechnol J. 2016 Sep 13;15(2):227–36. doi: 10.1111/pbi.12608 (PMC5258864; doi:10.1111/pbi.12608)
Supplement: Supplementary file 1 — Figure S1 T‐DNA elements of pPTN 809D5 and pASTA binary vectors. Figure S2 Southern blot analysis on selected pPTN809D soya bean events. Figure S3 Northern blot analyses on four‐gene stack soya bean lineage derived from SDA event (535‐9) × 824‐1 event (pPTN809D). Figure S4 Phenotypic coloration of soya bean seeds and derived oil from selected pASTA events. Figure S5 Southern blot analyses on selected transgenic soya bean events (pASTA). Figure S6 Northern blot analyses on selected immature T2 generation embryos obtained from selected pASTA soya bean events. Figure S7 Astaxanthin and β‐carotene levels in selected transgenic soya bean (pASTA) seed grown under greenhouse conditions. Figure S8 Astaxanthin and ß‐carotene levels in selected transgenic soya bean (pASTA) seed grown under field conditions. Figure S9 Tocopherol and tocotrienol levels in selected transgenic soya bean (pASTA) seed grown under greenhouse conditions. Table S1 Aquafeed test formulations with incremental SPC inclusion rates Table S2 Amino acid profiles of aquafeed SPC test formulations Table S3 Fatty acid profile of Kampachi flesh fed diets with increasing percentage of SDA Table S4 Fatty acid profiles on T1 seed derived from pPTN809D5 Table S5 Fatty acid profiles on F1 populations Table S6 Fatty acid profiles on F3 seeds producing EPA Table S7 Relative percentage of carotenoids in seed of selected transgenic soya bean events Table S8 Fatty acid profiles on selected orange colour F1 seeds producing ALA, SDA or EPA Table S9 Fatty acid profiles, total oil and carotenoid content on selected orange colour F2 seeds producing EPA Table S10 Fatty acid profiles and carotenoid content of mature soya bean seed from field harvest (2012) Table S11 Fatty acid profiles and carotenoid content of mature soya bean seed from field harvest (2013) [file PBI-15-227-s001.docx]

Towards the development of a sustainable soybean-based feedstock for aquaculture

Hyunwoo Park^1,2^, Steven Weier^3^ Fareha Razvi^2^, Pamela A. Peña^1,2^, Neil A. Sims^4^, Jennica Lowell^4^, Cory Hungate^4^, Karma Kissinger^4^, Gavin Key^4^ Paul Fraser^5^, Johnathan A. Napier^6^, Edgar B. Cahoon^2,7^ and Tom E. Clemente^1,2,*^

1- Department of Agronomy & Horticulture, University of Nebraska-Lincoln, Lincoln, NE USA

2- Center for Plant Science Innovation, University of Nebraska-Lincoln, Lincoln, NE USA

3-Department of Food Science and Technology, The Food Processing Center, University of Nebraska-Lincoln, Lincoln, NE USA

4-Kampachi Farms, LLC, Kona, HI USA

5- Centre for Systems and Synthetic Biology, School of Biological Sciences, Royal Holloway, University of London, Egham, Surrey, TW20 OEX, UK

6- Department of Biological Chemistry, Rothamsted Research, Harpenden, Herts AL5 2JQ, UK

7- Department of Biochemistry, University of Nebraska-Lincoln, Lincoln, NE USA

*- Correspondence (Tel 402-472-1428; email (tclemente1@unl.edu)

**Supporting Information: Tables and figures**

| Supporting Table 1: Aquafeed test formulations with incremental SPC inclusion rates | | | | | | |
| --- | --- | --- | --- | --- | --- | --- |
| Ingredient | 0% SPC | 10% SPC | 20% SPC | 30% SPC | 40% SPC | 100% SPC |
| Pre-ground blend | 97.22 | 78.98 | 60.25 | 40.81 | 21.36 | 0.00 |
| Profine VF (SPC) | 0.00 | 11.71 | 23.63 | 35.78 | 48.15 | 73.61 |
| Potato Starch | 0.00 | 1.75 | 3.54 | 4.75 | 5.51 | 6.51 |
| Fish, HFPC | 0.00 | 1.51 | 3.48 | 5.51 | 7.54 | 0.00 |
| Squid meal | 0.00 | 1.2 | 2.19 | 3.21 | 4.23 | 0.00 |
| Blood meal SD | 0.00 | 1.92 | 3.63 | 5.56 | 7.33 | 13.89 |
| Soy lecithin | 1.74 | 1.76 | 1.77 | 1.79 | 1.81 | 1.84 |
| Vitamin Pre-mix | 0.58 | 0.59 | 0.59 | 0.6 | 0.6 | 0.61 |
| Stay C-35% | 0.07 | 0.07 | 0.07 | 0.07 | 0.07 | 0.07 |
| Choline Cl 60% | 0.00 | 0.07 | 0.17 | 0.25 | 0.35 | 0.36 |
| Mineral Mix F-1 | 0.29 | 0.29 | 0.3 | 0.3 | 0.3 | 0.31 |
| Ca phosphate | 0.00 | 0.00 | 0.00 | 0.72 | 1.81 | 1.84 |
| L-lysine | 0.06 | 0.04 | 0.02 | 0.01 | 0.01 | 0.01 |
| MHA 84% | 0.00 | 0.01 | 0.15 | 0.31 | 0.46 | 0.47 |
| Ethoxquin | 0.02 | 0.02 | 0.02 | 0.02 | 0.02 | 0.02 |
| Mold inhibitor | 0.02 | 0.02 | 0.02 | 0.02 | 0.02 | 0.02 |
| Pre-ground blend consisted of: 67% fish meal (sardine 71.6/7.8), potato starch 16%, fish hydrolyzed fish protein concentrate (HFPC) 10% (74.6/8), squid meal 5% (85.2/3.6), and blood meal 2%. Numbers with each column refer to ingredient percentage of formulation. | | | | | | |

| Supporting Table 2: Amino acid profiles of aquafeed SPC test formulations | | | | | | |
| --- | --- | --- | --- | --- | --- | --- |
| Amino Acid | 0% SPC | 10% SPC | 20% SPC | 30% SPC | 40% SPC | 100% SPC |
| Tryptophan | 0.53 | 0.57 | 0.58 | 0.63 | 0.69 | 0.81 |
| Cysteine | 0.34 | 0.38 | 0.41 | 0.44 | 0.47 | 0.55 |
| Methionine | 1.00 | 0.96 | 0.88 | 0.81 | 0.75 | 0.62 |
| Aspartic acid | 3.59 | 3.96 | 4.14 | 4.38 | 4.92 | 5.60 |
| Threonine | 1.59 | 1.65 | 1.66 | 1.68 | 1.79 | 1.83 |
| Serine | 1.53 | 1.62 | 1.74 | 1.90 | 2.15 | 2.36 |
| Glutamic acid | 5.12 | 5.52 | 5.98 | 6.50 | 7.30 | 8.18 |
| Proline | 1.96 | 2.13 | 2.12 | 2.27 | 2.28 | 2.36 |
| Glycine | 2.45 | 2.40 | 2.31 | 2.34 | 2.52 | 2.21 |
| Alanine | 2.38 | 2.37 | 2.30 | 2.32 | 2.42 | 2.44 |
| Valine | 2.07 | 2.21 | 2.24 | 2.26 | 2.50 | 2.80 |
| Isoleucine | 1.67 | 1.71 | 1.74 | 1.73 | 1.84 | 0.57 |
| Leucine | 3.05 | 3.20 | 3.34 | 3.52 | 3.88 | 4.42 |
| Tyrosine | 1.10 | 1.18 | 1.22 | 1.22 | 1.33 | 1.47 |
| Phenylalanine | 1.61 | 1.79 | 1.88 | 2.01 | 2.22 | 2.59 |
| Lysine | 2.97 | 2.99 | 3.09 | 3.17 | 3.48 | 3.55 |
| Histidine | 1.12 | 1.18 | 1.22 | 1.34 | 1.49 | 1.80 |
| Arginine | 2.51 | 2.46 | 2.75 | 2.82 | 3.25 | 3.40 |
| Amino acid levels are reported in percentage of whole sample. Analyses were conducted at Eurofins Inc. Des Moines, IA. Note: percentages of glutamic acid and aspartic acid, include those of glutamine and asparagine, respectively. | | | | | | |

| Supporting Table 3**:** Fatty acid profile of Kampachi flesh fed diets with increasing percentage of SDA | | | | | | | | | | | | |
| --- | --- | --- | --- | --- | --- | --- | --- | --- | --- | --- | --- | --- |
| **SDA ratio** | **14:0** | **16:0** | **16:1** | **18:0** | **18:1** | **18:2** | **GLA** | **ALA** | **SDA** | **ETA** | **EPA** | **DHA** |
| 90:10 | 1.3±0.2C | 12.5±0.3C | 2.8±0.4C | 4.5±0.3A | 22.9±1.2A | 11.1±0.3A | 7.4±0.5A | 12.7±0.7A | 10.2±0.8A | 1.1±0.3 | 2.8±0.1C | 3.2±0.3B |
| 75:25 | 2.0±0.1B | 13.7±0.7B | 4.0±0.2B | 3.8±0.4B | 22.8±1.3A | 10.8±0.3A | 6.4±0.3B | 11.2±0.4B | 9.0±0.4B | 1.0±0.1 | 3.7±0.1B | 3.9±0.5B |
| 50:50 | 3.3±0.2A | 15.1±0.2A | 6.0±0.2A | 3.8±0.3B | 20.5±0.6B | 8.7±0.4B | 4.4±0.2C | 8.5±0.2C | 6.9±0.2C | 1.2±0.0 | 6.0±0.1A | 6.3±0.5A |
| SDA ratio column indicates the oil blend coating. 90:10, 75:25 and 50:50 refers to the ratio of STA soybean oil to fish oil coated on to the SPC-40T formulation. Numbers within the respective columns are means percentage of the respective fatty acid ± SD. Means were tabulated from flesh samples harvested from nine fish per ratio. Feeding trial was conducted for 78 days. Abbreviations for fatty acids are as follows: 14:0- myristic acid, 16:0- palmitic acid, 16:1- palmitoleic acid, 18:0- stearic acid, 18:1- oleic acid, 18:2- linoleic acid, GLA- gamma-linolenic caid, ALA- alpha-linolenic acid, SDA- stearidonic acid, ETA- eicosatetraenoic acid, EPA- eicosapentaenoic acid, and DHA-docosahexaenoic acid. Columns with letters indicate significant differences among the means (p≤0.01) as differentiated by Tukey’s multiple comparison test. | | | | | | | | | | | | |
|  | | | | | | | | | | | | |

| Supporting Table 4: Fatty acid profiles on T_1_ seed derived from pPTN809D5 | | | | | | | | |
| --- | --- | --- | --- | --- | --- | --- | --- | --- |
| Event | 16:0 | 18:0 | 18:1 | 18:2 | 18:3 | 20:1 | 20:2 | 20:3 |
| WT (Thorne) | 11.9±0.4 | 2.6±0.1 | 14.5±2.2 | 51.7±1.1 | 17.3±2.1 | 0±0 | 0±0 | 0±0 |
| 824-1 | 10.4±1.1 | 3.4±0.9 | 14.8±4.8 | 52.0±4.3 | 9.3±2.0 | 0.8±0.5 | 1.8±1.6 | 0.5±0.4 |
| 824-8 | 9.7±0.6 | 5.3±0.5 | 12.1±1.9 | 43.2±2.9 | 10.8±1.0 | 2.5±0.7 | 6.0±1.3 | 2.2±0.4 |
| 826-4 | 10.4±0.4 | 3.3±0.6 | 13.8±2.0 | 54.0±2.6 | 14.0±1.7 | 0.7±0.7 | 1.3±1.8 | 0.5±0.7 |
| Numbers within the respective fatty acid columns are the mean percentage of the fatty acid±SD. A total of 10 to 39 positive  cotyledon chips were used in calculation of the means, Note; Null recessive T_1_ seed were not used in the calculations. The  abbreviations for fatty acids are as follows 16:0, palmitic acid; 18:0, stearic acid; 18:1, oleic acid; 18:2, linoleic acid; 18:3,  linolenic acid; 20:1, eicosenoic acid; 20:2, eicosadienoic acid; 20:3, eicosatrienoic acid. | | | | | | | | |

| Supporting Table 5: Fatty acid profiles on F_1_ populations | | | | | | | | | | | | | | |
| --- | --- | --- | --- | --- | --- | --- | --- | --- | --- | --- | --- | --- | --- | --- |
| Event/Stack | 16:0 | 18:0 | 18:1 | 18:2 | GLA | ALA | SDA | 20:1 | 20:2 | UN | ARA | 20:3 | ETA | EPA |
| WT (Thorne) | 12.2 | 3.8 | 15.5 | 51.1 | 0.0 | 13.3 | 0.0 | 0.0 | 0.0 | 0.0 | 0.0 | 0.0 | 0.0 | 0.0 |
| 824-1 (T_2_) | 11.1 | 4.8 | 9.6 | 49.6 | 0.0 | 10.6 | 0.0 | 1.7 | 4.7 | 0.0 | 0.0 | 1.2 | 0.0 | 0.0 |
| 824-1 (T_2_) | 10.9 | 3.9 | 11.8 | 50.5 | 0.0 | 11.3 | 0.0 | 0.8 | 1.5 | 0.0 | 0.0 | 0.6 | 0.0 | 0.0 |
| 824-8 (T_2_) | 9.7 | 5.2 | 9.7 | 43.5 | 0.0 | 11.3 | 0.0 | 2.2 | 6.4 | 0.0 | 0.0 | 2.4 | 0.0 | 0.0 |
| SDA | 9.7 | 2.8 | 15.7 | 1.9 | 2.8 | 14.1 | 41.3 | 0.0 | 0.0 | 0.0 | 0.0 | 0.0 | 0.0 | 0.0 |
| 824-8 X SDA | 12.0 | 4.1 | 11.3 | 12.2 | 3.2 | 22.0 | 17.4 | 0.6 | 0.2 | 1.5 | 0.5 | 0.7 | 7.3 | 2.5 |
| 824-8 X SDA | 12.0 | 3.8 | 9.0 | 4.5 | 3.9 | 23.6 | 22.2 | 0.4 | 0.1 | 1.8 | 0.7 | 0.9 | 10.9 | 3.6 |
| SDA X 824-8 | 12.4 | 3.2 | 7.2 | 3.3 | 3.2 | 25.4 | 23.5 | 0.3 | 0.1 | 1.7 | 0.7 | 1.0 | 11.3 | 4.3 |
| SDA X 824-8 | 10.6 | 4.3 | 4.8 | 5.9 | 5.3 | 23.7 | 21.5 | 0.4 | 0.2 | 2.5 | 0.9 | 0.7 | 10.8 | 4.4 |
| 824-1 X SDA | 11.3 | 3.0 | 9.2 | 6.1 | 3.1 | 38.3 | 13.3 | 0.6 | 0.2 | 1.5 | 0.6 | 1.5 | 5.7 | 2.5 |
| 824-1 X SDA | 11.8 | 3.1 | 10.6 | 10.1 | 1.4 | 47.7 | 6.0 | 0.5 | 0.2 | 0.7 | 0.2 | 1.1 | 2.4 | 1.2 |
| 824-1 X SDA | 10.9 | 3.6 | 8.7 | 5.0 | 3.6 | 30.0 | 17.8 | 1.0 | 0.3 | 2.0 | 0.6 | 2.4 | 8.2 | 2.6 |
| SDA X 824-1 | 11.5 | 3.1 | 12.2 | 6.6 | 2.5 | 37.7 | 11.7 | 0.8 | 0.2 | 1.3 | 0.5 | 1.7 | 5.1 | 2.3 |
| Event/Stack column indicates the parental events used for crossing. SDA refers to event 535-9. Numbers within the respective fatty acid columns  are the percentage of the respective fatty acid. Single positive cotyledon chips were used in the calculation. The abbreviations for fatty acids are as follows GLA, γ-linoleic acid; ALA, α-linoleic acid; SDA, stearidonic acid; ARA, arachidonic acid; ETA, eicosatetraenoic aicd; EPA, eicosapentaenoic acid; UN, unknown fatty acid. | | | | | | | | | | | | | | |

| Supporting Table 6: Fatty acid profiles on F_3_ seeds producing EPA | | | | | | | | | | | | | | |  |
| --- | --- | --- | --- | --- | --- | --- | --- | --- | --- | --- | --- | --- | --- | --- | --- |
| Stack | 16:0 | 18:0 | 18:1 | 18:2 | GLA | ALA | SDA | 20:1 | 20:2 | UN | ARA | 20:3 | ETA | EPA | |
| **824-8 X SDA** | 12.8±0.2 | 3.9±0.7 | 11.5±3.1 | 5.1±0.6 | 4.9±2.6 | 22.8±3.9 | 20±1.0 | 0.6±0.1 | 0.2±0.1 | 2.4±1.1 | 0.6±0.1 | 1.4±0.7 | 8.8±0.3 | 2.6±0.7 | |
| **824-8 X SDA** | 13.9±0.7 | 4.2±0.3 | 8.4±1.0 | 3.3±0.5 | 2.8±0.6 | 16.4±1.5 | 27.0±1.7 | 0.4±0.0 | 0.4±0.1 | 1.3±0.2 | 0.5±0.1 | 0.8±0.0 | 12.8±0.5 | 5.1±0.8 | |
| **824-8 X SDA** | 10.9±0.3 | 3.7±0.4 | 11.7±1.9 | 4.7±1.0 | 2.6±0.8 | 33.2±3.6 | 18.8±3.0 | 0.9±0.2 | 0.2±0.1 | 1.0±0.3 | 0.2±0.1 | 2.1±0.6 | 6.6±1.5 | 1.6±0.5 | |
| **824-8 X SDA** | 11.7±0.2 | 3.5±0.2 | 11.2±2.0 | 9.0±3.3 | 1.2±1.1 | 43.1±10.7 | 9.7±6.7 | 0.4±0.1 | 0.1±0.0 | 0.6±0.6 | 0.2±0.2 | 0.9±0.0 | 4.6±3.8 | 1.9±0.9 | |
| **824-8 X SDA** | 12.3±0.6 | 3.2±0.1 | 15.3±2.5 | 4.1±0.5 | 2.0±0.6 | 27.4±4.2 | 19.6±3.1 | 0.5±0.2 | 0.3±0.1 | 0.9±0.3 | 0.2±0.1 | 1.4±0.4 | 8.3±2.0 | 2.4±0.8 | |
| **SDA X 824-1** | 12.3±0.8 | 3.7±0.5 | 9.4±1.2 | 9.9±7.6 | 8.7±6.4 | 23.1±13.1 | 14.7±9.1 | 0.6±0.2 | 0.4±0.4 | 4.5±3.4 | 1.4±1.0 | 0.8±0.2 | 6.8±4.3 | 2.1±1.0 | |
| **SDA X 824-1** | 12.1±0.4 | 3.5±0.2 | 13.8±2.8 | 5.8±2.6 | 3.7±1.4 | 28.3±7.8 | 17.4±6.2 | 0.8±0.2 | 0.1±0.1 | 1.7±0.6 | 0.4±0.1 | 1.0±0.3 | 7.8±2.9 | 2.0±0.5 | |
| **SDA X 824-1** | 11.9±0.5 | 3.5±0.5 | 10.7±2.0 | 5.9±3.0 | 3.9±4.4 | 33.5±11.0 | 16.5±5.8 | 0.6±0.2 | 0.1±0.1 | 1.7±2.0 | 0.5±0.5 | 1.2±0.5 | 6.5±2.2 | 1.9±0.4 | |
| F_3_ seed derived from respective F_1_ seed from Supplemental Table 5. Numbers within the respective fatty acid columns are mean percentage of the fatty acid±SD. 3 to 10 positive cotyledon chips were used to calculate means. UN, unknown fatty acid. | | | | | | | | | | | | | | |  |

| Supporting Table 7: Relative percentage of carotenoids in seed of selected transgenic soybean events | | | | | | | | | |
| --- | --- | --- | --- | --- | --- | --- | --- | --- | --- |
|  | WT  (Thorne) | 806-14 | 806-11 | 818-1 | 806-4 | 807-5 | 822-1 | 817-3 | 806-3 |
| Alpha carotene | 0 | 9.4 | 3.9 | 6 | 0 | 2.1 | 2.6 | 8.9 | 7.5 |
| Lutein | 100 | 0.6 | 0.1 | 0.7 | 2.7 | 0.5 | 0.4 | 0.5 | 0.8 |
| Beta carotene | 0 | 90 | 84.8 | 86.2 | 85.6 | 90.3 | 91.4 | 80 | 91.7 |
| Echinenone | 0 | 0 | 2.3 | 1.4 | 1.9 | 1.6 | 1.2 | 1.7 | 0 |
| Canthaxanthin | 0 | 0 | 3 | 1.6 | 4.8 | 1.5 | 1 | 2.7 | 0 |
| Phenioxanthin | 0 | 0 | 3.4 | 2 | 4 | 2 | 1.4 | 2.9 | 0 |
| Astaxanthin | 0 | 0 | 2.5 | 2.1 | 1 | 2 | 2 | 3.3 | 0 |
| Total | 100 | 100 | 100 | 100 | 100 | 100 | 100 | 100 | 100 |
| Phy | - | + | + | + | + | + | + | + | + |
| Crtz | - | - | + | + | + | - | + | + | + |
| Crtw | - | - | + | + | + | + | + | + | - |
| The columns display the relative percentages of the respective carotenoids within seed derived from selected transgenic events (pASTA). The +/- designations across the phy, crtz, and crtw rows indicate the presence (+) or absence (-) of the corresponding transgene as determined by molecular analyses on the corresponding transgenic event. | | | | | | | | | |

| Supporting Table 8: Fatty acid profiles on selected orange color F_1_ seeds producing ALA, SDA or EPA | | | | | | | | | | | | | | | |
| --- | --- | --- | --- | --- | --- | --- | --- | --- | --- | --- | --- | --- | --- | --- | --- |
| Stack | 16:0 | 18:0 | 18:1 | 18:2 | GLA | ALA | SDA | 20:1 | 20:2 | UN | ARA | 20:3 | ETA | EPA | Phenotype |
| ETP X 818-3 | 10.51 | 2.69 | 9.98 | 8.95 | 0.00 | 51.85 | 0.00 | 0.00 | 0.00 | 0.00 | 0.00 | 0.00 | 0.00 | 0.00 | ALA/Car |
| ETP X 818-3 | 11.21 | 3.91 | 11.23 | 9.91 | 4.88 | 28.15 | 11.22 | 1.28 | 0.89 | 2.97 | 0.85 | 2.04 | 4.62 | 0.42 | EPA/Car |
| ETP X 818-3 | 10.85 | 3.02 | 8.40 | 5.76 | 5.87 | 27.57 | 28.52 | 0.00 | 0.00 | 0.00 | 0.00 | 0.00 | 0.00 | 0.00 | SDA/Car |
| ETP X 807-2 | 9.79 | 3.30 | 16.73 | 13.92 | 4.94 | 27.84 | 7.94 | 1.64 | 1.11 | 2.63 | 0.58 | 2.14 | 3.20 | 0.84 | EPA/Car |
| ETP X 807-2 | 9.82 | 2.73 | 12.88 | 9.92 | 8.24 | 32.08 | 21.78 | 0.00 | 0.00 | 0.00 | 0.00 | 0.00 | 0.00 | 0.00 | SDA/Car |
| ETP X 807-2 | 10.27 | 2.84 | 13.51 | 13.60 | 0.00 | 55.81 | 0.00 | 0.00 | 0.00 | 0.00 | 0.00 | 0.00 | 0.00 | 0.00 | ALA/Car |
| ETP X 807-2 | 10.88 | 3.43 | 10.58 | 8.02 | 4.48 | 32.92 | 13.31 | 0.92 | 0.44 | 2.10 | 0.63 | 2.00 | 5.27 | 1.51 | EPA/Car |
| ETP X 807-2 | 10.21 | 3.14 | 12.58 | 10.23 | 3.77 | 34.03 | 11.10 | 0.71 | 0.38 | 1.76 | 0.65 | 1.82 | 4.73 | 1.71 | EPA/Car |
| 807-2 X ETP | 9.86 | 2.76 | 11.51 | 14.27 | 0.00 | 58.79 | 0.00 | 0.00 | 0.00 | 0.00 | 0.00 | 0.00 | 0.00 | 0.00 | ALA/Car |
| 806-14 X ETP | 9.70 | 2.55 | 19.24 | 11.53 | 5.50 | 33.93 | 14.78 | 0.00 | 0.00 | 0.00 | 0.00 | 0.00 | 0.00 | 0.00 | SDA/Car |
| 806-14 X ETP | 9.95 | 2.51 | 17.57 | 14.93 | 0.00 | 51.41 | 0.00 | 0.00 | 0.00 | 0.00 | 0.00 | 0.00 | 0.00 | 0.00 | ALA/Car |
| 806-14 X ETP | 9.45 | 3.30 | 16.29 | 10.99 | 4.01 | 29.77 | 10.34 | 1.35 | 0.65 | 1.95 | 0.44 | 2.64 | 4.33 | 1.09 | EPA/Car |
| 806-14 X ETP | 9.76 | 2.46 | 14.47 | 11.25 | 6.95 | 32.39 | 19.02 | 0.00 | 0.00 | 0.00 | 0.00 | 0.00 | 0.00 | 0.00 | SDA/Car |
| Stack column indicates the parental events used in crosses. ETP refers to soybean stack 824-1 X 535-9. Events 818-3, 807-2 and 806-14 are carotenoid  producing soybean used to create gene stack in soybean. Numbers within the respective fatty acid columns are the percentage of the fatty  acid. Single positive cotyledon chips were used in the calculation. UN refers to unknown fatty acid observed in GC trace. Phenotype column summarizes  the omega-3 fatty acid present and synthesis of carotenoid (Car) as indicated by seed color. | | | | | | | | | | | | | | | |

| Supporting Table 9: Fatty acid profiles, total oil and carotenoid content on selected orange color F_2_ seeds producing EPA | | | | | | | | | | | | |
| --- | --- | --- | --- | --- | --- | --- | --- | --- | --- | --- | --- | --- |
| Event/Stack | 16:0 | 18:0 | 18:1 | 18:2 | GLA | ALA | SDA | ETA | EPA | total oil | astaxanthin | β-carotene |
| WT (Thorne) | 11.8±0.8 | 3.6±0.2 | 15.8±2.2 | 53.9±1.6 | 0.0±0.0 | 12.9±0.9 | 0.0±0.0 | 0.0±0.0 | 0.0±0.0 | 22.2±1.1 | 0.0±0.0 | 0.0±0.0 |
| 806-14 | 10.9±0.2 | 3.2±0.2 | 18.6±3.5 | 52.6±1.8 | 0.0±0.0 | 12.8±2.1 | 0.0±0.0 | 0.0±0.0 | 0.0±0.0 | 20.3±2.1 | 0.0±0.0 | 1834.8±264.6 |
| 818-3 | 11.8±0.6 | 3.4±0.1 | 16.9±1.7 | 51.8±0.9 | 0.0±0.0 | 14.2±1.7 | 0.0±0.0 | 0.0±0.0 | 0.0±0.0 | 20.3±0.7 | 19.0±3.2 | 931.2±5.8 |
| 807-2 | 11.1±0.4 | 3.3±0.1 | 18.0±1.8 | 51.6±1.0 | 0.0±0.0 | 14.0±1.1 | 0.0±0.0 | 0.0±0.0 | 0.0±0.0 | 18.2±0.8 | 38.7±1.5 | 1751.6±98.4 |
| ETP | 12.2±0.6 | 3.4±0.3 | 10.2±1.3 | 11.9±7.4 | 7.3±5.8 | 29.1±13.2 | 11.5±7.1 | 4.7±3.1 | 1.6±0.7 | 21.8±0.3 | N/D | N/D |
| ETP | 11.8±0.6 | 3.6±0.2 | 16.6±4.9 | 5.3±1.6 | 3.8±1.0 | 27.8±7.7 | 17.2±4.4 | 6.6±1.7 | 1.5±0.2 | 22.7±0.9 | N/D | N/D |
| 806-14 X ETP | 10.1±0.5 | 3.9±0.1 | 13.7±3.3 | 12.2±4.2 | 5.2±6.6 | 35.8±16.7 | 8.1±3.3 | 3.3±1.5 | 1.0±0.2 | 18.7±0.0 | 0.0±0.0 | 1440.7±182.3 |
| ETP X 818-3 | 12.0±0.7 | 3.9±0.4 | 12.6±1.9 | 11.7±5.7 | 5.4±5.3 | 31.7±11 | 10.3±5.3 | 4.2±2.2 | 1.3±0.6 | 21.4±1.2 | 25.8±4.1 | 396.9±8.8 |
| ETP X 807-2 | 10.2±0.7 | 2.9±0.5 | 22.4±3.7 | 18.9±11.2 | 3.6±3.4 | 29.1±14.4 | 5.0±3.6 | 2.0±1.5 | 0.7±0.4 | 17.7±0.7 | 26.8±2.7 | 833.0±23.8 |
| ETP X 807-2 | 10.4±0.4 | 3.2±0.6 | 23.2±7.8 | 18.0±11.9 | 2.3±1.6 | 28.4±6.5 | 5.6±3.4 | 2.5±1.6 | 1.0±0.5 | 17.3±0.5 | 44±6.9 | 1139.4±181.5 |
| ETP X 807-2 | 9.6±0.8 | 3.5±0.5 | 15.7±6.9 | 23.0±12.5 | 4.2±3.1 | 28.4±12.3 | 5.5±5.5 | 2.4±2.5 | 0.9±0.5 | 14.4±0.3 | 23±1.0 | 527±46.7 |
| Numbers within the respective fatty acid columns are mean percentage of the fatty acid, total oil content (%) and carotenoid content (µg/g). Three to 8 positive  seeds were used in calculation of the means for fatty acid profiles. Positive seeds from each parental events and stacks were collected respectively and  ground together and then the soybean powder was used for total oil and carotenoid analyses. Two replicates were used in calculation of the means for total  oil and carotenoid content. ETP refers to soybean stack 824-1 X 535-9 F_3_ population (N/D implies Not Determined). | | | | | | | | | | | | |

| Supporting Table 10: Fatty acid profiles and carotenoid content of mature soybean seed from field harvest (2012) | | | | | | | | | | | | |  |  |
| --- | --- | --- | --- | --- | --- | --- | --- | --- | --- | --- | --- | --- | --- | --- |
| Event/Stack | 16:0 | 18:0 | 18:1 | 18:2 | GLA | ALA | SDA | ARA | ETA | EPA | Astaxanthin | β-carotene | | |
| WT (Thorne) | 11.5±0.3 | 3.9±0.2 | 20.0±0.0 | 55.0±0.6 | 0.0±0.0 | 9.0±0.4 | 0.0±0.0 | 0.0±0.0 | 0±0 | 0±0 | 0±0 | 0±0 | | |
| 806-14 | 9.6±0.3 | 5.3±0.3 | 17.5±0.2 | 56.9±0.0 | 0.0±0.0 | 10.2±0.1 | 0.0±0.0 | 0.0±0.0 | 0±0 | 0±0 | 0±0 | 2228.8±207.9 | | |
| 818-3 | 10.5±0.0 | 3.9±0.2 | 18.3±1.4 | 57.2±0.3 | 0.0±0.0 | 9.6±0.9 | 0.0±0.0 | 0.0±0.0 | 0±0 | 0±0 | 14.8±6.9 | 1113.5±227.4 | | |
| 807-2 | 9.6±0.0 | 6.5±0.2 | 15.0±1.9 | 56.2±0.3 | 0.0±0.0 | 12.0±1.8 | 0.0±0.0 | 0.0±0.0 | 0±0 | 0±0 | 45.5±0.4 | 930.8±0 | | |
| ETP | 10.8±0.8 | 3.3±0.2 | 15.9±3.7 | 4.1±2.6 | 3.3±1.1 | 27.7±7.0 | 20.0±6.6 | 0.3±0.1 | 7.2±2.8 | 2.1±0.9 | N/D | N/D | | |
| ETP | 11.0±0.8 | 3.2±0.3 | 16.4±2.6 | 8.3±3.0 | 3.9±1.8 | 32.4±7.1 | 13.7±6.4 | 0.3±0.1 | 4.5±1.9 | 1.4±0.3 | N/D | N/D | | |
| 806-14 X ETP | 9.0±0.7 | 3.7±0.3 | 16.3±3.3 | 14.7±3.3 | 9.3±7.4 | 24.7±14.3 | 8.8±4.3 | 1.0±0.8 | 3.6±1.7 | 1.3±0.5 | 0±0 | 2129.6±753.5 | | |
| ETP X 818-3 | 9.5±0.5 | 3.0±0.1 | 16.4±3.8 | 7.8±1.2 | 1.2±0.2 | 47.3±4.5 | 8.0±1.7 | 0.2±0.2 | 2.6±0.5 | 1.0±0.1 | 9.4±4.0 | 1248.6±560.7 | | |
| ETP X 807-2 | 9.0±0.4 | 3.2±0.2 | 17.8±1.6 | 16.7±5.6 | 0.7±0.2 | 43.9±5.0 | 3.6±0.7 | 0.3±0.4 | 1.3±0.2 | 0.6±0.1 | 12.6±8.9 | 1512.0±334.9 | | |
| ETP X 807-2 | 9.4±0.6 | 3.1±0.1 | 15.4±2.6 | 14.3±5.2 | 4.9±2.9 | 31.9±8.3 | 10.0±4.3 | 0.5±0.3 | 3.9±1.9 | 1.4±0.6 | 22.2±11.2 | 1574.2±186.6 | | |
| Numbers within the respective fatty acid columns are mean percentage of the fatty acid and carotenoid content (µg/g). Two to 29 positive seeds  were used per replication in calculation of the means for fatty acid profiles. Positive seeds from each parental events and stacks (F_3_) were collected, ground and the soybean powder was used for carotenoid analysis. Two replicates were used in calculation of the means for carotenoid content. ETP refers to soybean stack 824-1 X 535-9 F_4_ population (N/D implies Not Determined). | | | | | | | | | | | | | |  |

| Supporting Table 11: Fatty acid profiles and carotenoid content of mature soybean seed from field harvest (2013) | | | | | | | | | | | |  |
| --- | --- | --- | --- | --- | --- | --- | --- | --- | --- | --- | --- | --- |
| Event/Stack | 16:0 | 18:0 | 18:1 | 18:2 | GLA | ALA | SDA | ARA | ETA | EPA | Astaxanthin | ß-carotene |
| WT (Thorne) | 12.2±0.3 | 3.7±0.2 | 19.1±3.9 | 52.4±2.4 | 0.0±0.0 | 11.1±1.8 | 0.0±0.0 | 0.0±0.0 | 0±0 | 0±0 | 0±0 | 0±0 |
| 806-14 | 11.0±0.3 | 3.4±0.3 | 17.3±2.7 | 54.2±1.4 | 0.0±0.0 | 12.5±2.2 | 0.0±0.0 | 0.0±0.0 | 0±0 | 0±0 | 0±0 | 1415.6±25.1 |
| 818-3 | 11.7±0.4 | 3.3±0.1 | 18.1±2.4 | 53.8±1.0 | 0.0±0.0 | 11.7±1.1 | 0.0±0.0 | 0.0±0.0 | 0±0 | 0±0 | 17.5±1.6 | 843.0±90.3 |
| 807-2 | 11.1±0.5 | 3.8±0.4 | 18.3±3.8 | 51.5±2.5 | 0.0±0.0 | 13.8±1.5 | 0.0±0.0 | 0.0±0.0 | 0±0 | 0±0 | 34.0±4.2 | 747.4±4.2 |
| ETP | 12.1±0.9 | 3.9±0.2 | 12.2±3.3 | 3.2±1.4 | 3.9±0.7 | 23.7±2.3 | 21.9±3.6 | 0.5±0.1 | 9.4±1.7 | 3.0±1.0 | N/D | N/D |
| ETP | 11.6±0.7 | 3.6±0.2 | 11.5±2.5 | 6.3±2.7 | 4.3±1.5 | 32.4±7.1 | 15.9±5.8 | 0.5±0.1 | 5.9±2.3 | 1.9±0.5 | N/D | N/D |
| 806-14 X ETP | 12.0±1.3 | 4.5±0.5 | 16.4±2.9 | 18.9±6.6 | 9.9±4.1 | 17.7±4.4 | 7.0±2.9 | 1.3±0.3 | 2.9±1.3 | 1.1±0.4 | 0±0 | 1420.7±318.5 |
| ETP X 818-3 | 10.9±0.5 | 3.4±0.1 | 13.8±2.4 | 12.2±2.9 | 1.5±0.5 | 44.5±3.0 | 5.9±0.8 | 0.2±0.1 | 2.3±0.3 | 1.0±0.1 | 23.3±0.9 | 991.8±407.2 |
| ETP X 807-2 | 9.4±0.2 | 4.0±0.6 | 15.7±1.9 | 14.5±3.7 | 1.1±0.7 | 44.4±0.5 | 3.9±3.3 | 0.3±0.3 | 1.7±0.9 | 0.8±0.4 | 25.61±0.1 | 953.7±71.6 |
| ETP X 807-2 | 9.8±0.9 | 3.3±0.2 | 24.0±5.1 | 13.4±6.8 | 1.6±2.0 | 33.2±8.7 | 6.1±5.3 | 0.2±0.2 | 2.7±2.4 | 1.1±0.8 | 27.3±6.5 | 1150.0±112.8 |
| Numbers within the respective fatty acid columns are mean percentage of the fatty acid and carotenoid content (µg/g). Three to 10 positive seeds were used per replication in calculation of the means for fatty acid profiles. Positive seeds from each parental event and stack were collected, ground and the soybean powder was used for carotenoid analysis. Two replicates were used in calculation of the means for carotenoid content.  ETP refers to soybean stack 824-1 X 535-9 F_5_ population (N/D implies Not Determined). | | | | | | | | | | | | |


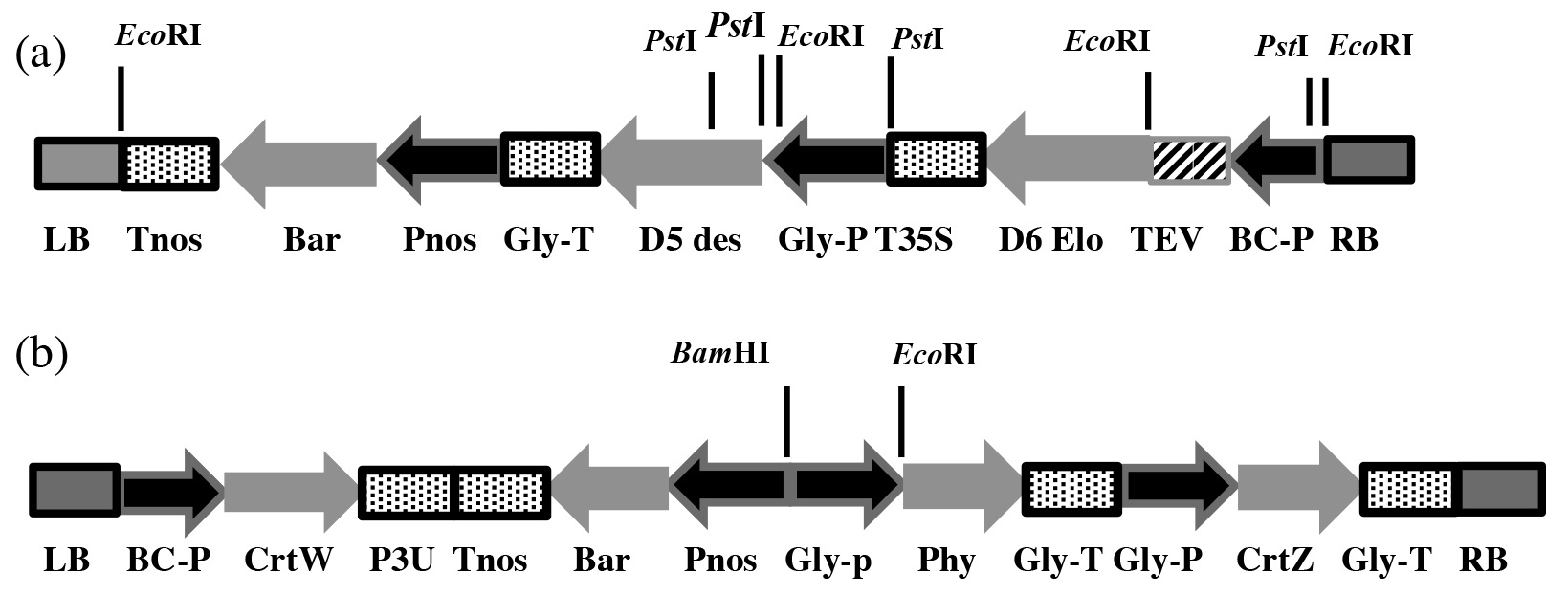


Supporting Figure 1: T-DNA elements of pPTN 809D5 and pASTA binary vectors.

Supporting Fig 1a: T-DNA element of pPTN809D5. Supporting Fig 1b: T-DNA element of pASTA. The abbreviations for the various genetic elements are as follows: BC-P, β-conglycinin promoter; TEV, tobacco etch translational enhancer; D6 Elo, Δ6 elongase; T35S, 35s terminator; Gly-p, Glycinin promoter; D5 des, Δ5 desaturase; Gly-T, glycinin terminator; Pnos, nopaline synthase promoter; Bar, bar gene; Tnos, nopaline synthase terminator; CrtW, β-carotene ketolase; Phy, phytoene synthase; CrtZ, β-carotene hydroxylase; P3U, phaseolin 3’UTR. RB and LB refer to the right border and left border elements.


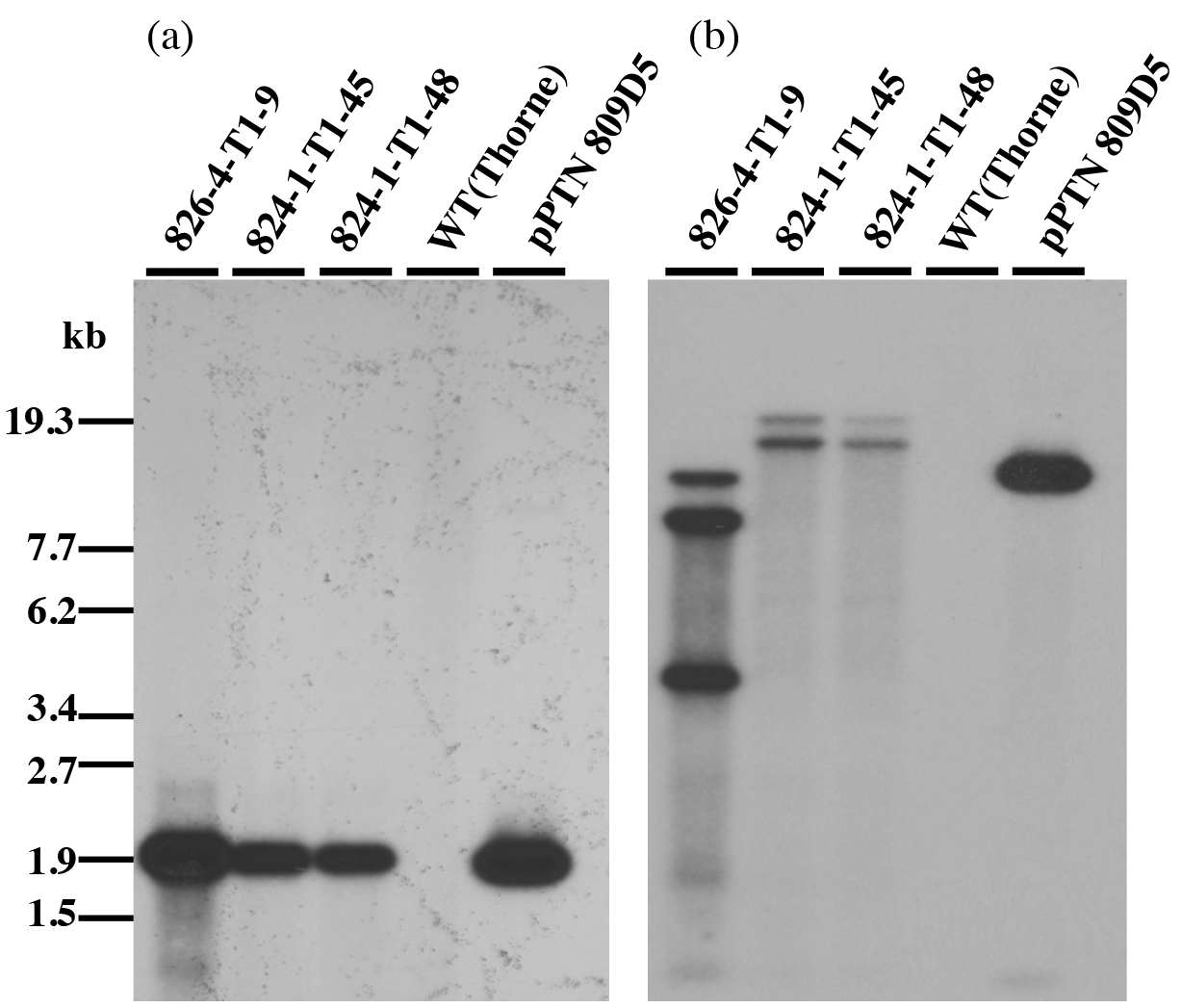


Supporting Figure 2: Southern blot analysis on selected pPTN809D soybean events

Total genomic DNA (10µg) digested with *Pst1* run per lane. Membrane hybridized with Δ6 desaturase (Supporting Fig 2a) and Δ5 desaturase (Supporting Fig 2b). WT (Thorne) lane refers to wild-type soybean genotype Thorne DNA. Lane designated pPTN 809D5 is 100 pg of plasmid DNA digested with *Pst*I.


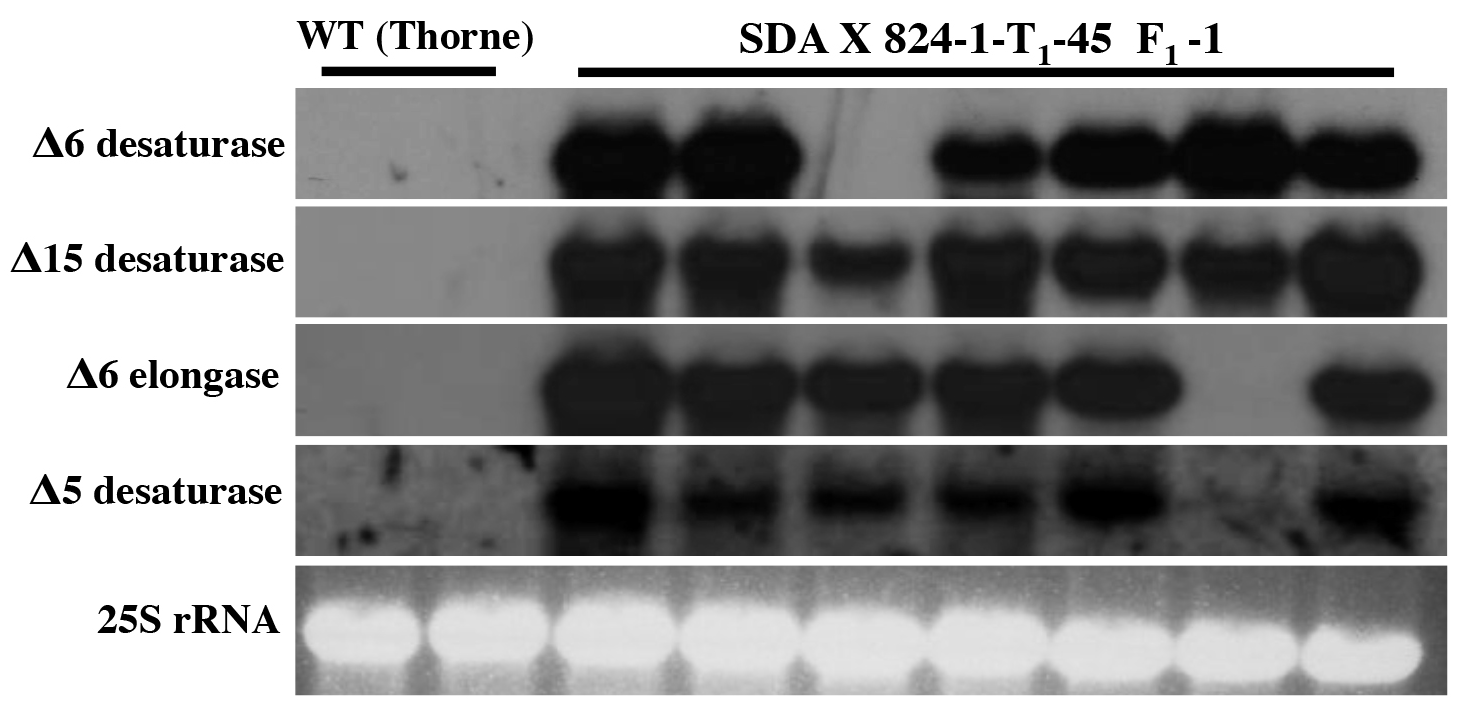


Supporting Figure 3: Northern blot analyses on four-gene stack soybean lineage derived from SDA event (535-9) x 824-1 event (pPTN809D)

Total RNA (15 µg) isolated from immature embryos run per lane. Lanes 1 & 2 represent total RNA isolated from control wild type Thorne immature embryos (WT (Thorne)). Lanes 3 through 7 refer to total RNA isolated from immature embryos (F_2_ generation) of the four-gene stack lineage. Top to bottom panels represent hybridization signals observed from Δ6 desaturase, Δ15 desaturase, Δ6 elongase and Δ5 desaturase gene probes, respectively. Bottom panel is 25S rRNA imaged from ∆5 desaturase gel, which mirrored lane loading on other gels.


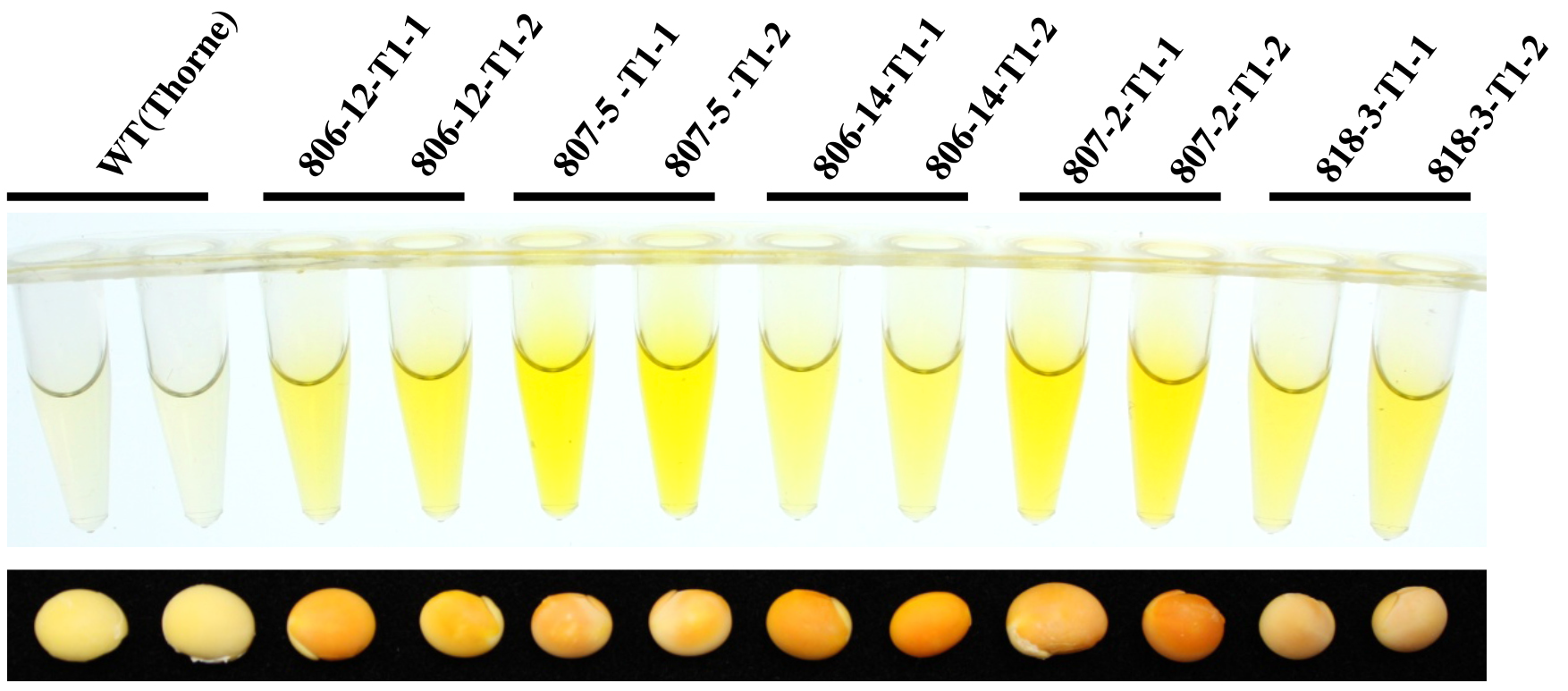


Supporting Figure 4: Phenotypic coloration of soybean seeds and derived oil from selected pASTA events

Seeds and derived oils from T_2_ generation of pASTA transgenic soybean events 806-12, expresses phytoene synthase and CrtZ; 807-5, expresses phyotene synthase and CrtW; 806-14, expresses only phytoene synthase; 807-2 and 818-3 express the three gene stack of pASTA. WT (Thorne) corresponds to control seed and derived oil.


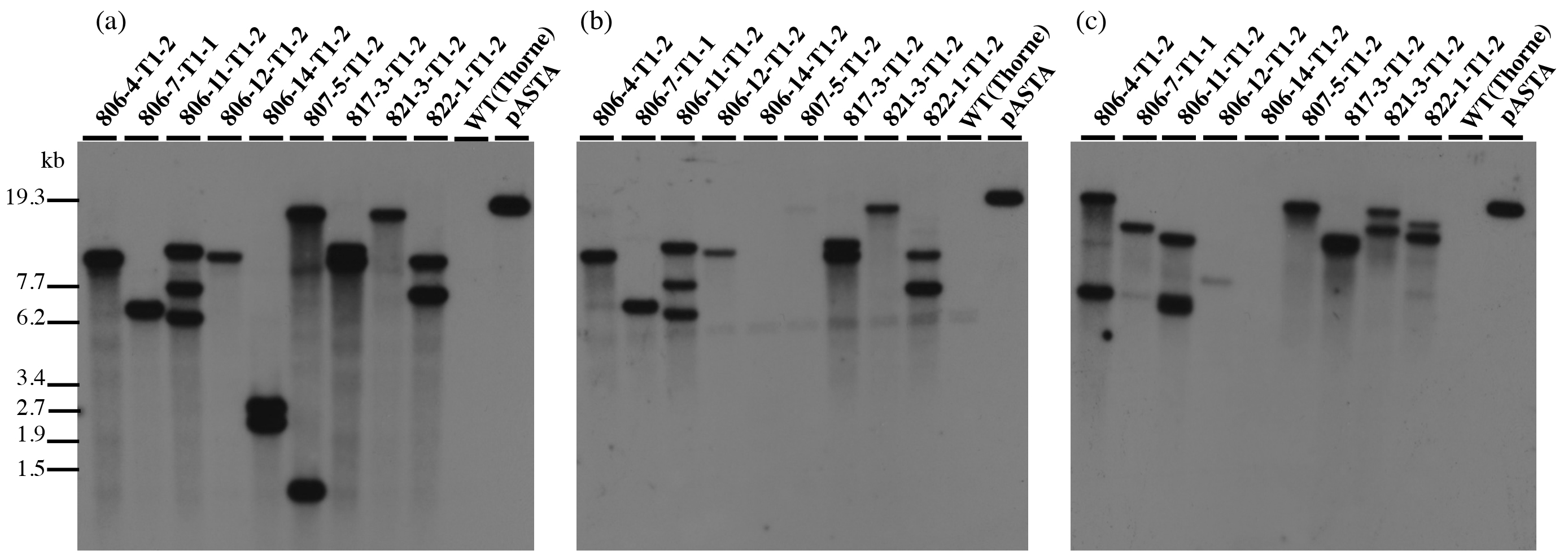


Supporting Figure 5: Southern blot analyses on selected transgenic soybean events (pASTA)

Total genomic DNA (10µg) digested with *Eco*RI and run per lane. DNA samples were isolated from the T_1_ individuals of the respective events. Blots were probed with phytoene synthase (Supporting Fig 5a), CrtZ (Supporting Fig 5b) and CrtW (Supporting Fig 5c). Lane 10 and 11 represent 10 µg DNA isolated wild type control Thorne (WT (Thorne)) and a 100pg of the binary plasmid pASTA digested with *Eco*RI*,* respectively.


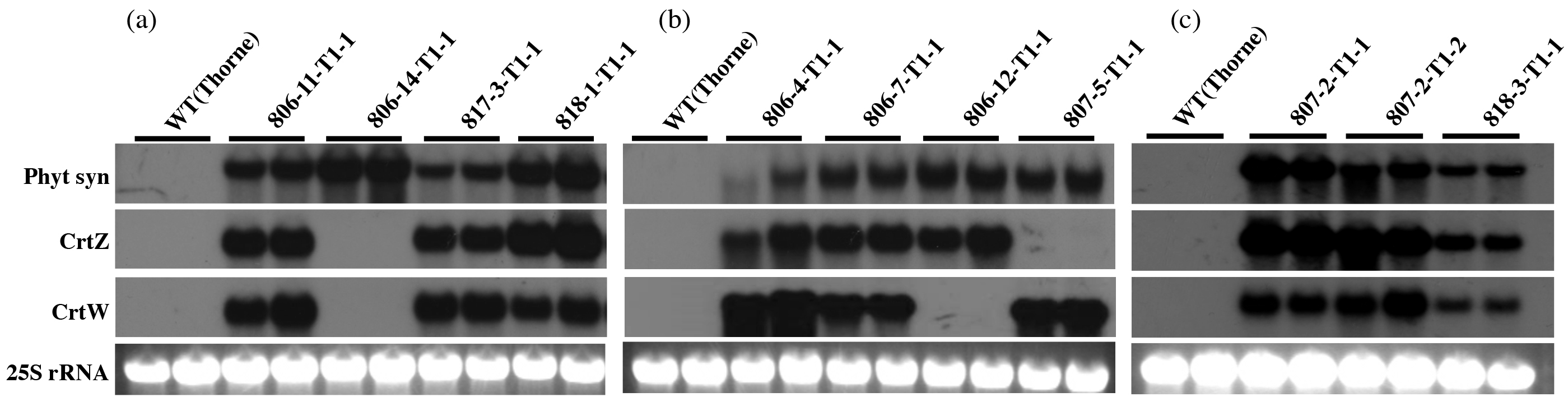


Supporting Figure 6: Northern blot analyses on selected immature T_2_ generation embryos obtained from selected pASTA soybean events.

Total RNA (15 µg) isolated from immature embryos run per lane. First two lanes within each panel represent total RNA isolated from control wild type Thorne immature embryos (WT (Thorne)). The remaining lanes within each panel represent RNA isolated from the respective transgenic event lineage, two T_2_ generation immature embryos from the same T_1_ plant, which displayed an observable colored phenotype. The three panels, top to bottom, across Fig S6a, S6b and S6c, represent hybridization signals from phytoene synthase (Phyt syn), Crtz and CrtW, respectively. Bottom panel is 25S rRNA imaged from CrtW gel, which mirrored lane loading on other gels.


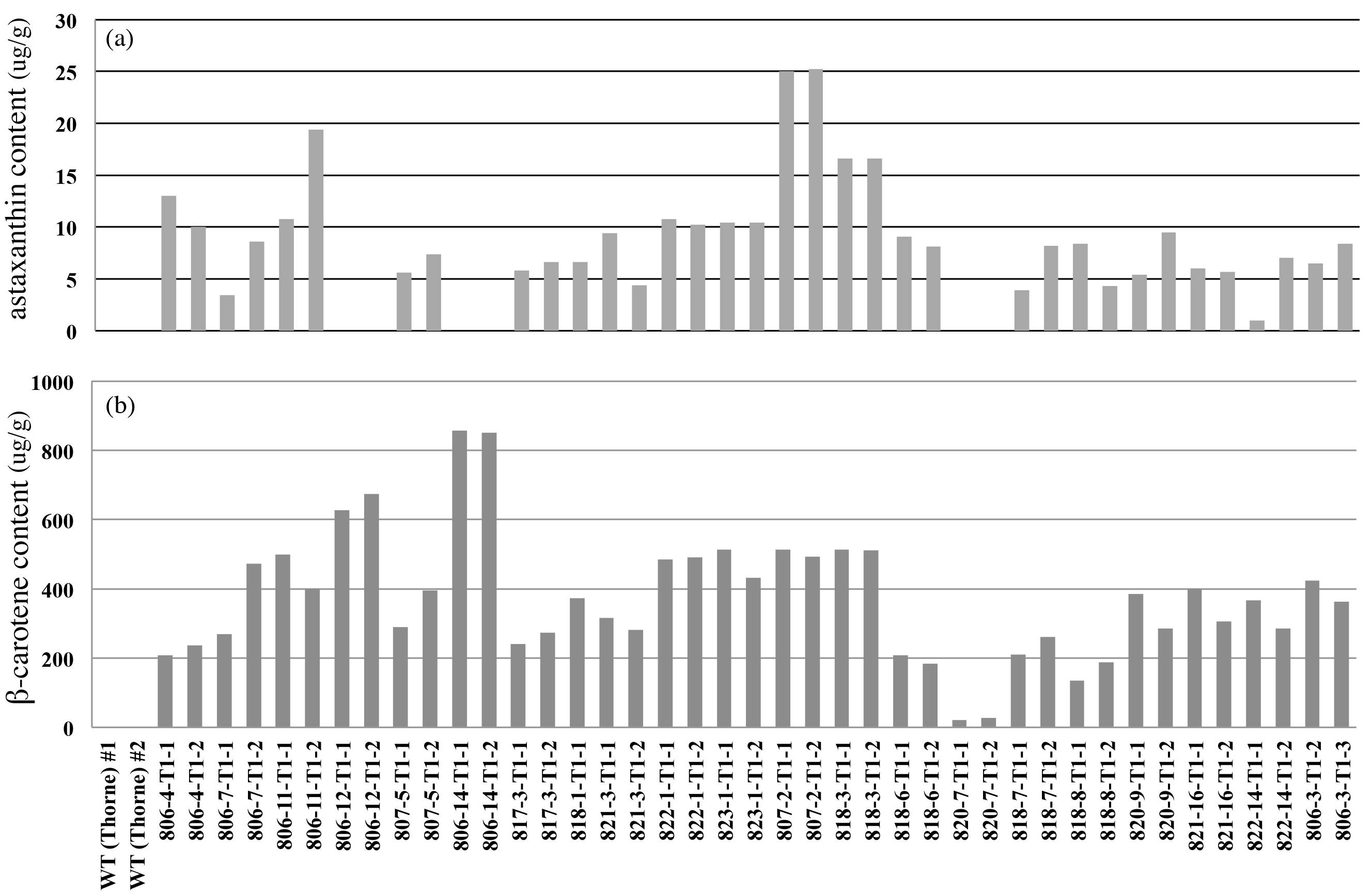


Supporting Figure 7: Astaxanthin and ß-carotene levels in selected transgenic soybean (pASTA) seed grown under greenhouse conditions

Oil samples from five T_2_ generation seed, which displayed an observable colored phenotype, were bulked extracted and carotenoid levels monitored via HPLC. Corresponding astaxanthin and ß-carotene levels (µg/gram sample) are shown in supplemental figure 7a and 7b, respectively. No detectable level of either carotenoid was observed in controls samples designated WT (Thorne).


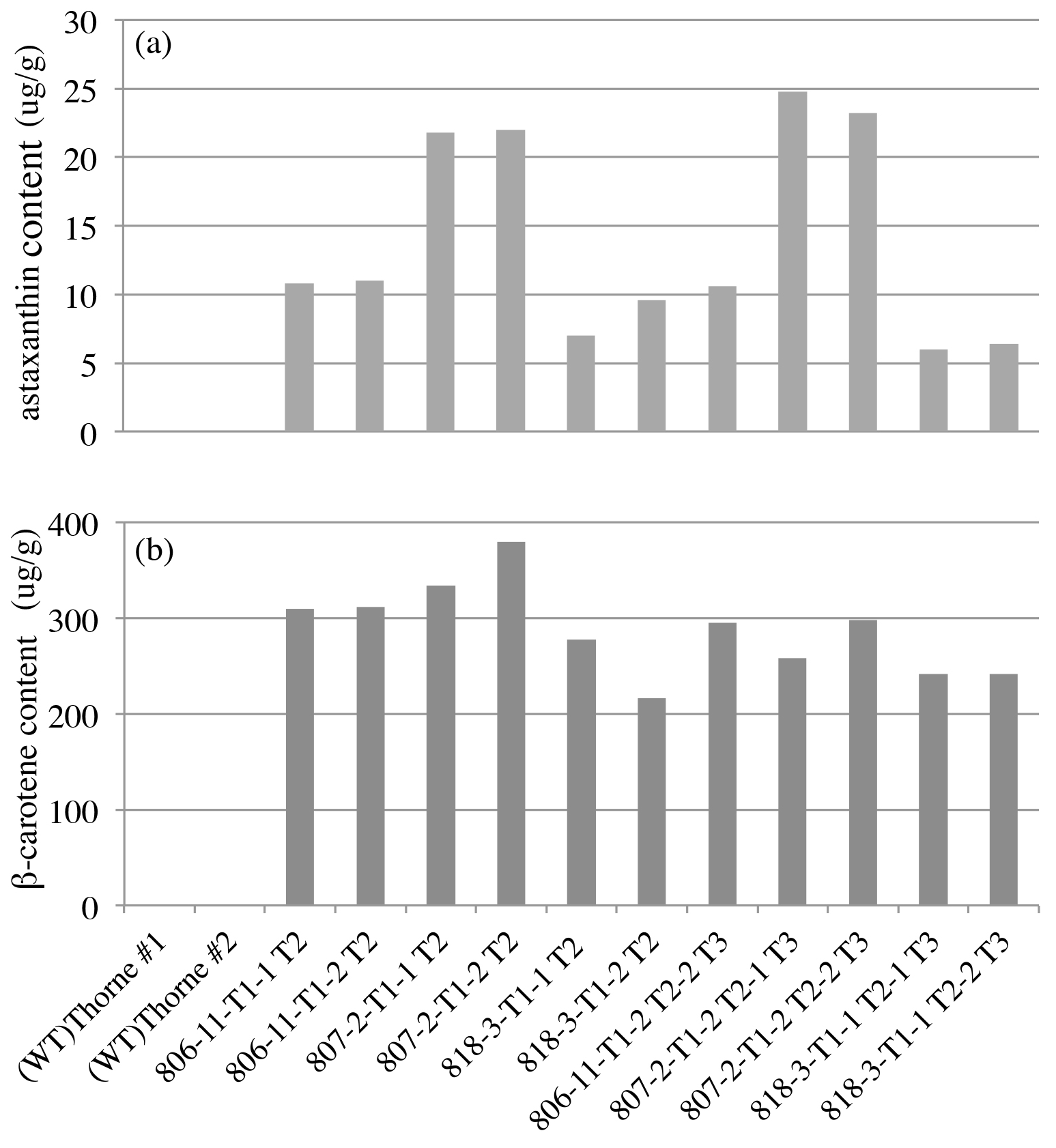


Supporting Figure 8: Astaxanthin and ß-carotene levels in selected transgenic soybean (pASTA) seed grown under field conditions

Oil samples from five T_4_ generation seed, which displayed an observable colored phenotype, were bulked extracted and carotenoid levels monitored via HPLC. Corresponding astaxanthin and ß-carotene levels (µg/gram sample) are shown in supporting figure 8a and 8b, respectively. No detectable level of either carotenoid was observed in controls samples designated WT (Thorne).


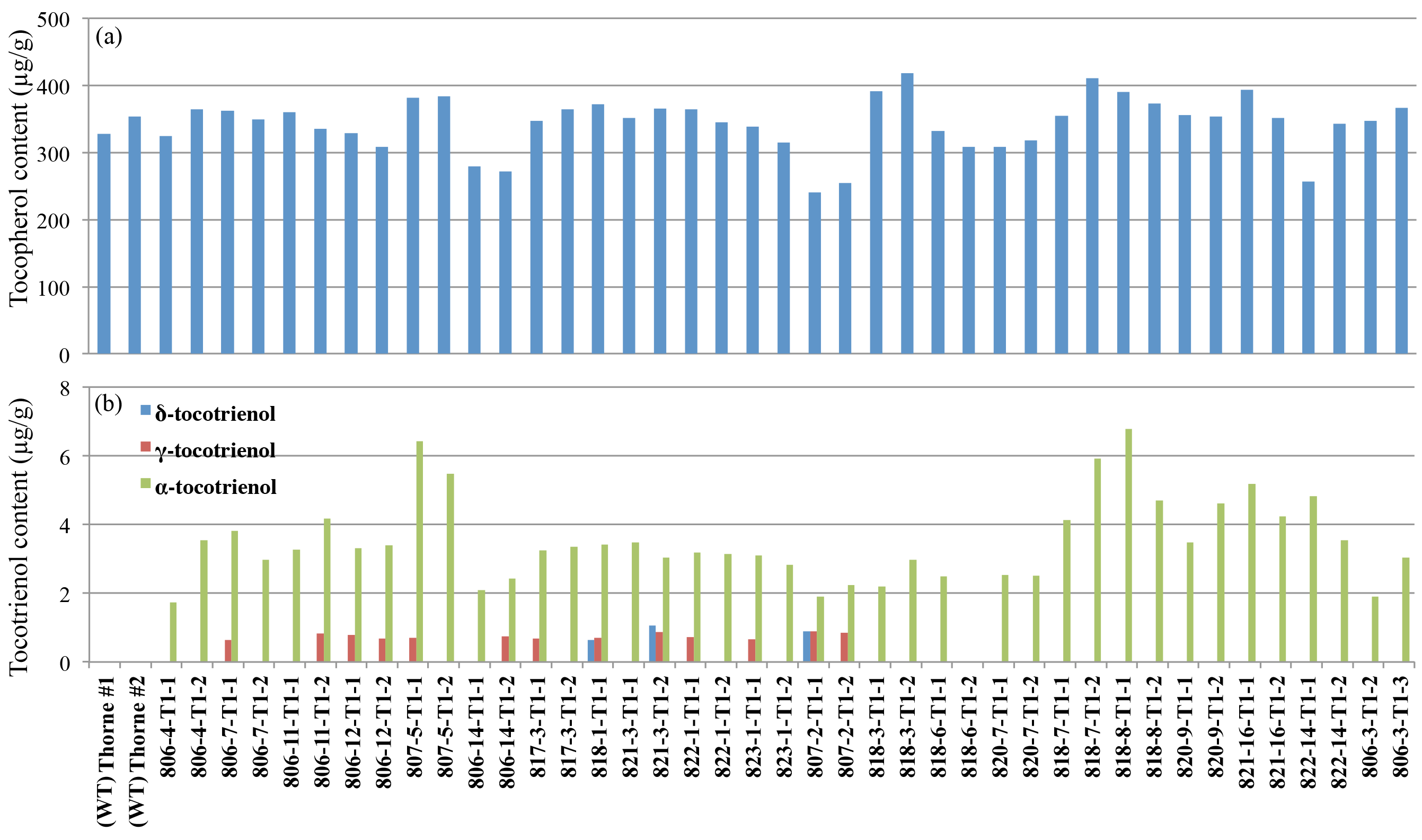


Supporting Figure 9: Tocopherol and tocotrienol levels in selected transgenic soybean (pASTA) seed grown under greenhouse conditions

Ground samples obtained from five T_2_ generation seed, which displayed an observable colored phenotype, was bulked extracted. Fifty mg of bulked powder was placed in a solution of ethanol:dichloromethane (9:1 v/v) and derived samples analyzed via HPLC. Corresponding tocopherol and tocotrienol levels (µg/gram sample) are shown in supporting figure 9a and 9b, respectively. Control samples designated WT (Thorne). Supporting Figure 9b bar designations are right to left, ∂-, γ− and α−tocotrienol. If only a single bar is present, indicates only α-tocotrienol detected.
